# Intratumoral Collagen Deposition Supports Angiogenesis Suggesting Anti‐angiogenic Therapy in Armored and Cold Tumors

**DOI:** 10.1002/advs.202409147

**Published:** 2025-01-17

**Authors:** Jie Mei, Kai Yang, Xinkang Zhang, Zhiwen Luo, Min Tian, Hanfang Fan, Jiahui Chu, Yan Zhang, Junli Ding, Junying Xu, Yun Cai, Yongmei Yin

**Affiliations:** ^1^ Department of Oncology The First Affiliated Hospital of Nanjing Medical University Nanjing Jiangsu 211166 P. R. China; ^2^ The First Clinical Medicine College Nanjing Medical University Nanjing Jiangsu 211166 P. R. China; ^3^ Department of Sports Medicine Huashan Hospital Affiliated to Fudan University Shanghai 200040 P. R. China; ^4^ Departments of Oncology Wuxi People's Hospital The Affiliated Wuxi People's Hospital of Nanjing Medical University Wuxi Medical Center Nanjing Medical University Wuxi Jiangsu 214023 P. R. China; ^5^ Departments of Gynecology The Women's Hospital Affiliated to Jiangnan University Wuxi 214023 China; ^6^ Central Laboratory Changzhou Jintan First People's Hospital The Affiliated Jintan Hospital of Jiangsu University Changzhou Jiangsu 213200 P. R. China; ^7^ Jiangsu Key Lab of Cancer Biomarkers Prevention and Treatment Collaborative Innovation Center for Personalized Cancer Medicine Nanjing Medical University Nanjing Jiangsu P. R. China

**Keywords:** anti‐angiogenic therapy, armored & cold tumor, collagen deposition, vascularization

## Abstract

A previous study classifies solid tumors based on collagen deposition and immune infiltration abundance, identifying a refractory subtype termed armored & cold tumors, characterized by elevated collagen deposition and diminished immune infiltration. Beyond its impact on immune infiltration, collagen deposition also influences tumor angiogenesis. This study systematically analyzes the association between immuno‐collagenic subtypes and angiogenesis across diverse cancer types. As a result, armored & cold tumors exhibit the highest angiogenic activity in lung adenocarcinoma (LUAD). Single‐cell and spatial transcriptomics reveal close interactions and spatial co‐localization of fibroblasts and endothelial cells. In vitro experiments demonstrate that collagen stimulates tumor cells to express vascular endothelial growth factor A (VEGFA) and directly enhances vessel formation and endothelial cell proliferation through sex determining region Y box 18 (SOX18) upregulation. Collagen inhibition via multiple approaches effectively suppresses tumor angiogenesis in vivo. In addition, armored & cold tumors display superior responsiveness to anti‐angiogenic therapy in advanced LUAD cohorts. Post‐immunotherapy resistance, the transformation into armored & cold tumors emerges as a potential biomarker for selecting anti‐angiogenic therapy. In summary, collagen deposition is shown to drive angiogenesis across various cancers, providing a novel and actionable framework to refine therapeutic strategies combining chemotherapy with anti‐angiogenic treatments.

## Graphical abstract

This study demonstrates that armored & cold tumors consistently exhibit elevated microvessel density levels across various cancer types. Mechanistically, collagen fosters angiogenesis by directly activating endothelial cells and increasing the expression and secretion of vascular endothelial growth factor A in tumor cells. Overall, this study offers a novel and robust perspective for the challenging decision‐making process regarding the selection of chemotherapy plus anti‐angiogenic therapy.

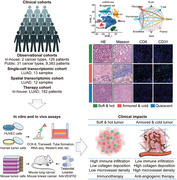

## 1 Introduction

The extracellular matrix (ECM) is a fundamental structural component of all tissues and organs, essential for the survival of multicellular organisms, including tumors.^[^ ^1^ ^]^ During tumor progression, collagen, a major ECM protein, plays a pivotal role in modulating the immune response within the tumor microenvironment (TME).^[^ ^2^ ^]^ Research has shown that both the quantity and structural arrangement of collagen influence immune cell infiltration in tumors.^[^ ^3^, ^4^ ^]^ Beyond immune modulation, collagen also governs tumor angiogenesis. As a critical ECM constituent, collagen provides structural support for vascular endothelial cells.^[^ ^5^, ^6^, ^7^ ^]^ However, the precise mechanisms by which collagen promotes angiogenesis remain unclear. Therefore, understanding the relationship between collagen and angiogenesis is crucial for elucidating tumor progression mechanisms and uncovering new therapeutic strategies.

In our previous study, tumors were classified into three subtypes based on collagen activity and immune signatures.^[^ ^8^ ^]^ Notably, the armored & cold subtype, defined as armored & cold tumors accompanied by high collagen activity and low immune infiltration, exhibited the poorest response to immune checkpoint blockade therapy. In addition, similar results also were observed by other research groups.^[^ ^9^, ^10^ ^]^ Furthermore, novel therapeutic targets were identified from these immuno‐collagenic subtypes, with stromal proteins such as B7‐H3 and vascularization markers being highly expressed in armored & cold tumors, suggesting their potential as therapeutic targets.^[^ ^8^ ^]^ In addition, anti‐B7‐H3 therapy could be effective for treating armored & cold tumors.^[^ ^11^ ^]^ However, the applicability of anti‐angiogenic therapy for these tumors remains uninvestigated.

Anti‐angiogenic therapy targets aberrant angiogenesis, a process where new blood vessels form to supply oxygen and nutrients to tissues.^[^ ^12^ ^]^ In tumors, however, angiogenesis is dysregulated, driving tumor progression and metastasis.^[^ ^13^, ^14^ ^]^ Anti‐angiogenic therapy aims to disrupt or inhibit this abnormal vascular growth, thereby restricting tumor blood supply and limiting its spread.^[^ ^15^ ^]^ Bevacizumab, the most widely used anti‐angiogenic agent, has been a staple in clinical practice for over 20 years.^[^ ^16^, ^17^ ^]^ Other common anti‐angiogenic drugs include Sorafenib, Sunitinib, and Endostatin.^[^ ^18^, ^19^ ^]^ While studies have linked immune and angiogenesis‐related factors to responses to anti‐angiogenic therapy,^[^ ^20^, ^21^ ^]^ reliable, predictive biomarkers remain lacking.

**Figure 1:**
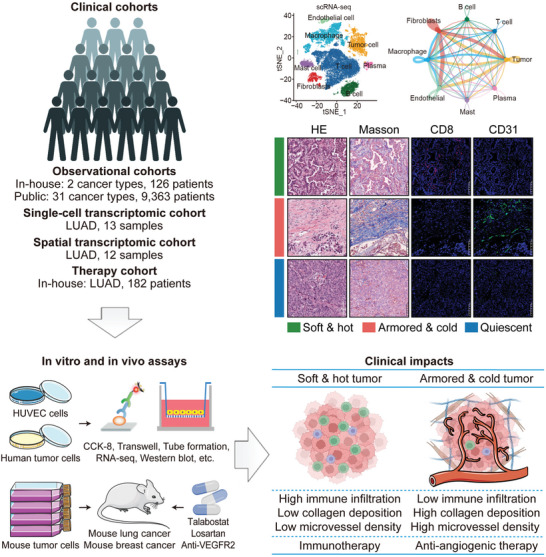
Schematic overview of the study. Clinical Cohorts and Analysis: Observational cohorts included the TCGA dataset (31 cancer types, 9363 patients) and two in‐house cohorts (2 cancer types, 126 patients), along with one LUAD single‐cell transcriptomic cohort (13 samples), one LUAD spatial transcriptomic cohort (12 samples), and one in‐house LUAD therapy cohort (182 patients). These cohorts were used to define the associations between different tumor subtypes and angiogenesis, explore the effects of collagen on angiogenesis, and assess the decision‐making impact of immuno‐collagenic subtypes. In vitro and in vivo assays: The effects of collagen on angiogenesis and the underlying mechanisms were investigated. Clinical effects: For refractory armored & cold tumors, anti‐angiogenic therapy proved more beneficial than immunotherapy, while for soft & hot tumors, immunotherapy was more advantageous than anti‐angiogenic therapy. Abbreviations: TCGA: The Cancer Genome Atlas; LUAD: lung adenocarcinoma; VEGFR2: vascular endothelial growth factor receptor 2; HE: hematoxylin and eosin; scRNA‐seq: single‐cell RNA‐sequencing. Copyrighted from the BioRender platform.

Given the strong association between our immuno‐collagenic subtypes and both immune and stromal markers,^[^ ^8^ ^]^ whether this classification could inform anti‐angiogenic therapy selection was examined. In addition, this study analyzed the relationship between immuno‐collagenic subtypes and angiogenesis using public and in‐house pan‐cancer cohorts. Additionally, the effects of collagen on angiogenesis were explored through in silico, in vitro, and in vivo models. An advanced lung adenocarcinoma (LUAD) cohort was included to assess how immuno‐collagenic subtypes impact therapeutic decision‐making (**Figure** **1**). Overall, our findings provide novel insights into collagen's role in angiogenesis and emphasize the potential of immuno‐collagenic subtypes as a guide for selecting anti‐angiogenic therapies.

## 2 Results

### 2.1 Armored & Cold Tumors Exhibited High Angiogenesis Activity and Poor Prognosis in LUAD

**Figure 2:**
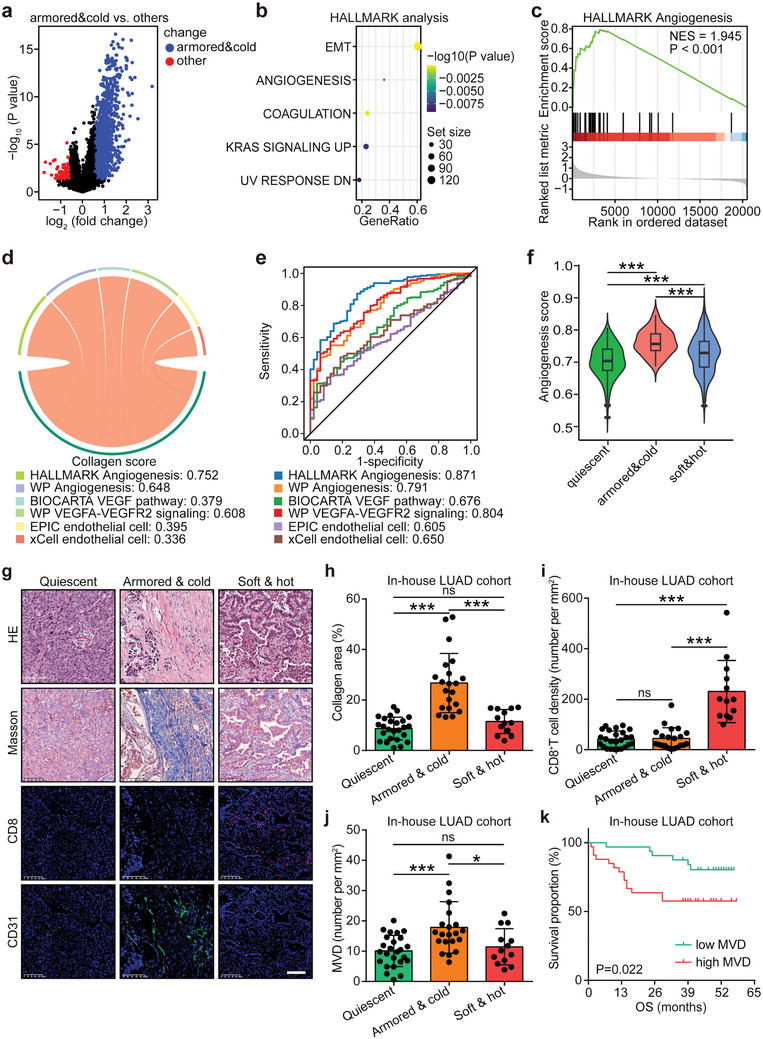
Armored & cold tumors exhibit high angiogenesis in LUAD. a) Volcano plot showing differentially expressed genes (DEGs) in armored & cold tumors compared to other subtypes in the TCGA‐LUAD cohort, with a threshold of *P*‐value < 0.05 and FC ≥ 1.5. b) HALLMARK analysis of upregulated genes in armored & cold tumors within the TCGA‐LUAD cohort. c) GSEA illustrating the correlation between the “HALLMARK Angiogenesis” signature and the armored & cold subtype in the TCGA‐LUAD cohort. d) Correlation between collagen score and multiple angiogenesis‐related features in the TCGA‐LUAD cohort, assessed using Pearson correlation. e) Discrimination values of six angiogenesis‐related features in distinguishing armored & cold tumors from other subtypes in the TCGA‐LUAD cohort. f) Differences in angiogenesis scores (average of six angiogenesis‐related features) across three immuno‐collagenic subtypes in the TCGA‐LUAD cohort, presented as mean ± SD. Statistical significance was determined using one‐way ANOVA with Tukey's multiple‐comparison test. ***P < 0.001. *n* (quiescent) = 214, *n* (armored & cold) = 48, *n* (soft & hot) = 242. g) Representative images of collagen, CD8, and CD31 expression in the three immuno‐collagenic subtypes in the in‐house LUAD cohort, with scale bar = 100 µm. h–j) Quantitative analysis of collagen, CD8, and CD31 expression in the three immuno‐collagenic subtypes in the in‐house LUAD cohort, shown as mean ± SD. Statistical significance was assessed using the Kruskal–Wallis test with Dunn's multiple‐comparison test for (h) and (i), and one‐way ANOVA with Tukey's multiple‐comparison test for (j). ns: non‐significant, *P < 0.05, ***P < 0.001. *n* (quiescent) = 25, *n* (armored & cold) = 21, *n* (soft & hot) = 13. k) Prognostic value of MVD in the in‐house LUAD cohort, with significance determined by the log‐rank test. *n* (low) = 32, *n* (high) = 33. Abbreviations: DEG: differentially expressed gene; TCGA: The Cancer Genome Atlas; LUAD: lung adenocarcinoma; FC: fold change; GSEA: gene set enrichment analysis; MVD: microvessel density; HE: hematoxylin and eosin; OS: overall survival; NES: normalized enrichment score.

To investigate distinct biological signatures in armored & cold tumors in LUAD, highly expressed genes were extracted (**Figure** **2**a) and analyzed using HALLMARK pathways. The analysis identified enrichment in epithelial–mesenchymal transition (EMT), angiogenesis, coagulation, and related processes (Figure 2b). Gene set enrichment analysis (GSEA) further associated the armored & cold phenotype with angiogenesis (Figure 2c). To characterize angiogenesis activity in tumor tissues, six angiogenesis‐related factors were evaluated: HALLMARK angiogenesis signature, WP angiogenesis signature, BIOCARTA VEGF pathway, WP VEGFA‐VEGFR2 signaling, endothelial cell abundance from the EPIC algorithm,^[^ ^22^ ^]^ and endothelial cell abundance from the xCell algorithm.^[^ ^23^ ^]^ Correlation analysis demonstrated significant positive relationships between the collagen score and these angiogenesis signatures (Figure 2d). In addition, these signatures showed high accuracy in distinguishing armored & cold tumors in LUAD (Figure 2e). An angiogenesis score was established by averaging the six angiogenesis‐related factors, revealing elevated expression in armored & cold tumors (Figure 2f).

High Intratumoral vessel abundance was validated in an in‐house LUAD cohort, aligning with previous findings.^[^ ^8^ ^]^ Samples exhibiting high levels of both tumor‐infiltrating immune cells (TIIC) and collagen were rare (Figure S1a, Supporting Information), and the armored & cold subtype was associated with the poorest prognosis (Figure S1b, Supporting Information). Furthermore, these tumors displayed high collagen expression and low CD8 levels (Figure 2g–i). Notably, microvessel density (MVD), determined via anti‐CD31 staining, was highest in armored & cold tumors (Figure 2g,j), and elevated MVD correlated with poor prognosis in the in‐house cohort (Figure 2k). Collectively, this integrative analysis underscores the overexpression of intratumoral vessels in armored & cold tumors, highlighting angiogenesis as a potential therapeutic target for this subtype.

### 2.2 Increased Intratumoral Collagen Deposition Enhanced the Angiogenesis Activity

**Figure 3:**
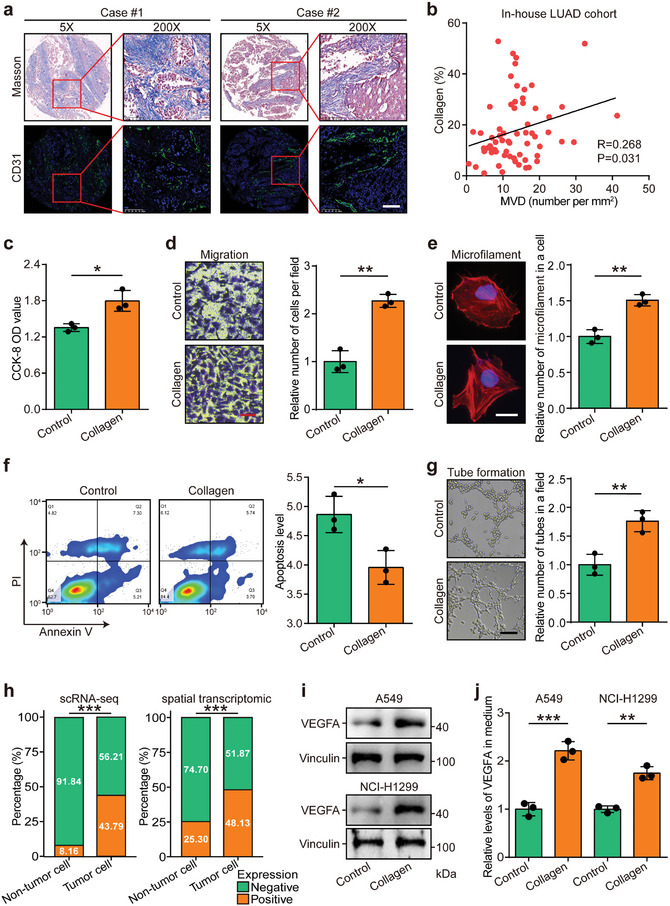
Intratumoral collagen deposition enhances endothelial cell activity. a) Representative images showing the distribution of collagen and CD31^+^ endothelial cells in the in‐house LUAD cohort. Scale bar = 100 µm. b) Correlation between collagen levels and MVD in LUAD samples. Significance was calculated using the Pearson correlation test. c–g) Detection of proliferation, migration, microfilament formation, apoptosis, and tube formation in control and collagen‐treated HUVEC cells in vitro. Quantitative analysis is presented as mean ± SD. Significance was calculated using the Student's *t*‐test. *P < 0.05, **P < 0.01. *n* = 3 per group. Scale bars: (d) and (g) = 100 µm, (e) = 10 µm. h) Expression of VEGFA in tumor and non‐tumor cells in the scRNA‐seq datasets (GSE131907 and GSE149655) and the spatial transcriptomic dataset (E‐MTAB‐13530). Significance was assessed using the Chi‐square test. ***P < 0.001. i) Detection of total VEGFA levels in control and collagen‐treated cancer cells by Western blotting. j) Detection of soluble VEGFA levels in the medium of control and collagen‐treated cancer cells by ELISA. Results were normalized to control values = 1. Data are presented as mean ± SD. Significance was assessed using the Student's *t*‐test. **P < 0.01, ***P < 0.001. *n* = 3 per group. Abbreviations: LUAD: lung adenocarcinoma; VEGFA: vascular endothelial growth factor A; HE: hematoxylin and eosin; scRNA‐seq: single‐cell RNA‐sequencing.

The correlation between collagen abundance and MVD was assessed in the in‐house LUAD cohort, revealing a positive association between increased collagen levels and MVD (**Figure** **3**a,b). To validate the stimulatory effects of collagen on angiogenesis, HUVEC cells were treated with collagen. This treatment significantly promoted cell proliferation, migration, and microfilament assembly, while inhibiting apoptosis (Figure 3c–f). Notably, collagen directly enhanced angiogenesis activity in HUVEC cells (Figure 3g). Vascular endothelial growth factor A (VEGFA), a key angiogenic factor in vascular endothelial cells,^[^ ^24^, ^25^ ^]^ was also analyzed. Subcellular localization studies using scRNA‐seq and transcriptomics data revealed that VEGFA was predominantly expressed in tumor cells (Figure 3h). As anticipated, collagen treatment increased both the expression and secretion of VEGFA in these cells (Figure 3i,j and Figure S2a, Supporting Information).

**Figure 4:**
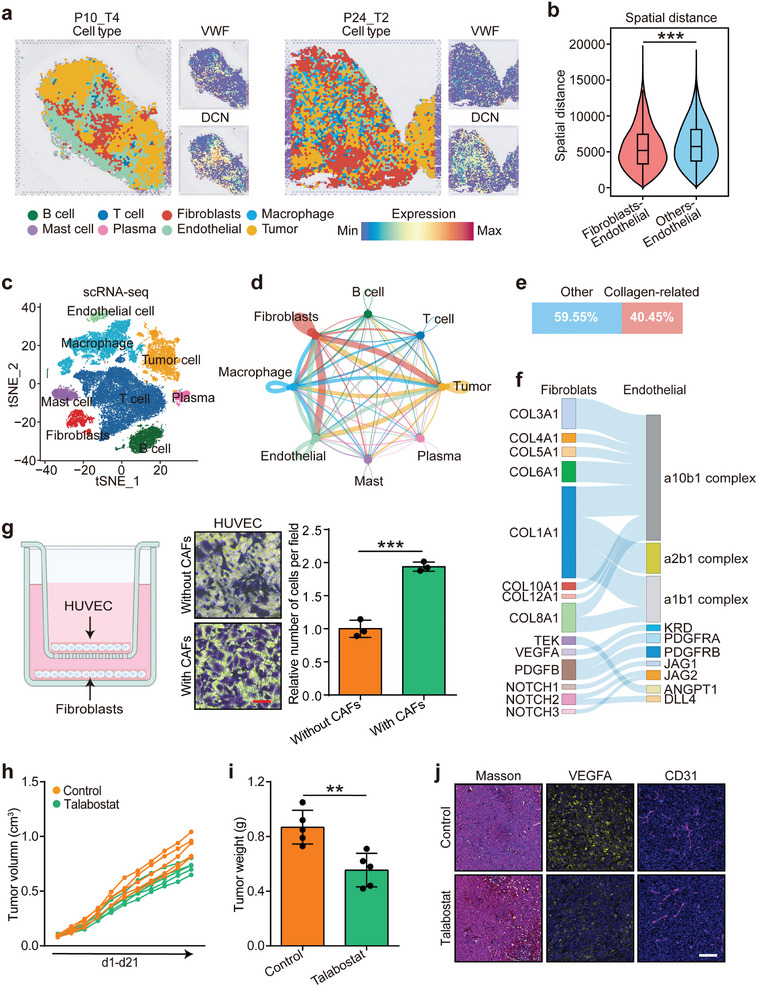
Spatial distributions and communications of fibroblasts and endothelial cells. a) Spatial cell charting of P10_T4 and P24_T2 LUAD samples obtained from the study by Marco De Zuani et al.^[^ ^26^ ^]^ VWF is a marker of endothelial cells and DCN is a marker of CAFs. b) Comparison of spatial distances between endothelial cells and fibroblasts or non‐fibroblasts in 12 LUAD samples. c) t‐SNE visualization of cell types annotated by established signatures in 13 patients with LUAD. d) The number of cell–cell communications among different cell types in the scRNA‐seq data of 13 patients with LUAD. The thickness of the lines represents the interaction frequency between subpopulations, as estimated using the CellPhoneDB tool. e,f) The collagen‐related pro‐angiogenic interactions between fibroblasts and endothelial cells. g) Schematic protocol illustrating the co‐culture of fibroblasts and endothelial cells and the effect of fibroblasts on the migration of endothelial cells. Data presented as mean ± SD. Statistical significance was calculated using the Student's *t*‐test. ***P < 0.001. *n* = 3 per group. Original magnification 200×, scale bar = 100 µm. h,i) Effects of Talabostat on tumor volume and tumor weight in C57BL/6 mice bearing Lewis cells. Data presented as mean ± SD. Significance was assessed using the Student's *t*‐test. **P < 0.01. *n* = 5 per group. j) The deposited levels of collagen and the expression levels of VEGFA and the endothelial cell marker CD31 in mouse tumor tissues. Original magnification 200×, scale bar = 100 µm. Abbreviations: LUAD: lung adenocarcinoma; VWF: von‐Willebrand factor; DCN: decorin; CAF: cancer‐associated fibroblast; VEGFA: vascular endothelial growth factor A; HE: hematoxylin and eosin; scRNA‐seq: single‐cell RNA‐sequencing.

Since intratumoral collagen is primarily synthesized by cancer‐associated fibroblasts (CAFs), the role of fibroblasts in modulating endothelial cell behavior was explored at the single‐cell level. Spatial transcriptomics data from 12 LUAD samples^[^ ^26^ ^]^ were analyzed, identifying six distinct cell types through unsupervised clustering (Figure S3a–e, Supporting Information). The results showed a tendency for endothelial cells to cluster around fibroblasts in spatial proximity (**Figures** **4**a and S4, Supporting Information). Further analysis of spatial distances between endothelial cells and other cell types confirmed that endothelial cells were positioned closer to fibroblasts than to other cell types (Figure 4b). To elucidate fibroblast‐endothelial cell interactions, a high‐resolution analysis was performed on 46108 cells from 13 patients with LUAD. These cells were categorized into eight cell types (Figure 4c and Figure S5a–d, Supporting Information), and a dissection of ligand–receptor interactions revealed that endothelial cells exhibited the most interactions with fibroblasts, more so than with any other cell type (Figure 4d–f and Figure S6a,b, Supporting Information). Importantly, these interactions were predominantly collagen‐related (Figure 4e,f), suggesting that fibroblast‐mediated angiogenesis activity in endothelial cells is driven by collagen. In co‐culture experiments, CAFs significantly enhanced the migration of HUVEC cells (Figure 4g). In vivo assays further validated these findings. Treatment with Talabostat, an inhibitor of fibroblast activation protein (FAP) that reduces collagen production,^[^ ^8^ ^]^ resulted in significant inhibition of tumor growth (Figure 4h,i). In addition, Talabostat reduced collagen deposition and inhibited the expression of VEGFA and CD31 in tumors (Figure 4j). Together, these results demonstrate that intratumoral collagen enhances angiogenesis by directly activating endothelial cells and promoting VEGFA expression, highlighting its potential as a therapeutic target in LUAD.

### 2.3 Collagen Promotes Angiogenesis of HUVEC Cells via Increasing SOX18 Expression

**Figure 5:**
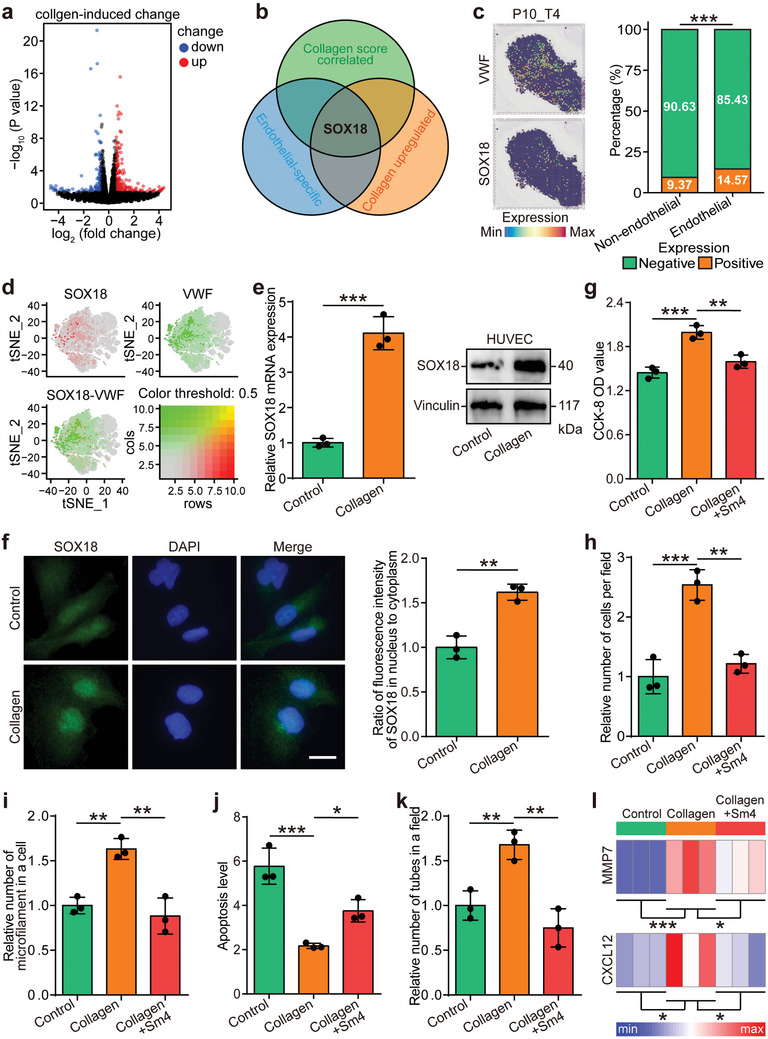
Collagen promotes angiogenesis via upregulating SOX18 in HUVEC cells. a) Volcano plot showing the differentially expressed genes (DEGs) in collagen‐treated HUVEC cells, with a threshold of *P*‐value < 0.05 and FC ≥ 1.5. b) Venn diagram illustrating the overlap between genes upregulated by collagen in HUVEC cells, genes positively correlated with the collagen score in the TCGA‐LUAD cohort, and endothelial‐specific genes identified in the scRNA‐seq dataset. c) Spatial charting of SOX18 and VWF expression in the P10_T4 LUAD sample from Marco De Zuani et al.’s study,^[^ ^26^ ^]^ alongside SOX18 expression in endothelial and non‐endothelial cells in the spatial transcriptomic dataset. Statistical significance was determined using the Chi‐square test. ***P < 0.001. d) *t*‐SNE visualization of SOX18 (gray to red) and VWF (gray to green) expression. e,f) Expression and subcellular localization of SOX18 in HUVEC cells, assessed by qPCR, Western blot, and immunofluorescence. Data are presented as mean ± SD. Significance was assessed using the Student's *t*‐test. ***P < 0.001. *n* = 3 per group. Scale bar = 5 µm. g–k) Detection of proliferation, migration, microfilament formation, apoptosis, and tube formation in control, collagen‐treated, and collagen/Sm4 co‐treated HUVEC cells in vitro. Data are presented as mean ± SD. Statistical significance was calculated using ANOVA with Tukey's multiple‐comparison test. *P < 0.05, **P < 0.01, ***P < 0.001. *n* = 3 per group. l) Expression of SOX18 downstream genes, MMP7 and CXCL12, in collagen‐treated and collagen/Sm4 co‐treated HUVEC cells. Statistical significance was assessed using ANOVA with Tukey's multiple‐comparison test. *P < 0.05, ***P < 0.001. *n* = 3 per group. Abbreviations: DEG: differentially expressed gene; TCGA: The Cancer Genome Atlas; LUAD: lung adenocarcinoma; FC: fold change; VWF: von‐Willebrand factor; SOX18: sex determining region Y box 18.

To investigate the mechanisms underlying collagen‐mediated angiogenesis, RNA sequencing was performed on collagen‐stimulated HUVEC cells. Collagen‐regulated genes in these cells were extracted (**Figure** **5**a), and the intersection of these genes with collagen‐correlated genes from the TCGA‐LUAD cohort and endothelial‐specific genes from the scRNA‐seq dataset was analyzed. SOX18, previously identified as a key regulator of angiogenesis,^[^ ^27^, ^28^ ^]^ emerged as a critical gene (Figure 5b). Further validation in transcriptomics data confirmed that sex determining region Y box 18 (SOX18) was highly expressed in endothelial cells (Figure 5c,d). Moreover, collagen stimulation was shown to upregulate SOX18 expression and promote its nuclear translocation (Figure 5e,f and Figure S2b, Supporting Information). The SOX18 inhibitor Sm4 significantly inhibited collagen‐induced increases in HUVEC cell proliferation, migration, microfilament assembly, and tube formation, while promoting apoptosis (Figure 5g–k). SOX18 is known to transcriptionally regulate several angiogenesis‐related genes, including CXCL12^[^ ^29^, ^30^ ^]^ and MMP7.^[^ ^31^, ^32^ ^]^ Sm4 treatment notably suppressed the collagen‐induced overexpression of CXCL12 and MMP7, confirming that collagen not only upregulates SOX18 expression but also enhances its transcriptional activity (Figure 5l).

**Figure 6:**
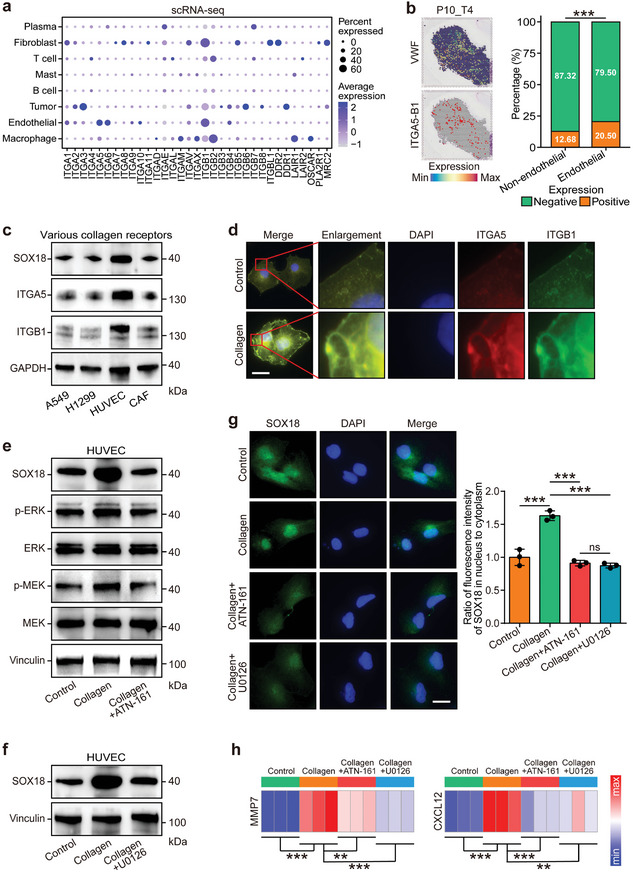
Collagen upregulates SOX18 expression through the integrin α5β1/MEK/ERK signaling pathway. a) Expression levels of collagen receptors across different cell types in the scRNA‐seq dataset, with ITGB1 and ITGA5 showing the highest positive correlation. b) Spatial charting of ITGA5‐B1 and VWF in the P10_T4 LUAD sample from Marco De Zuani et al.’s study,^[^ ^26^ ^]^ alongside ITGA5‐B1 expression in endothelial and non‐endothelial cells in the spatial transcriptomic dataset. Statistical significance was determined using the Chi‐square test. ***P < 0.001. c) Expression levels of SOX18, ITGA5, and ITGB1 in tumor cells (A549 and H1299), HUVECs, and CAFs. d) Enhanced co‐localization of ITGA5 and ITGB1 following collagen stimulation. Scale bar = 5 µm. e) Impact of collagen stimulation and ITGA5‐B1 blockade on the MEK/ERK pathway in HUVEC cells. f) Effects of collagen stimulation and MEK/ERK inhibition on SOX18 expression in HUVEC cells. g) Effects of collagen stimulation, ITGA5‐B1 blockade, and MEK/ERK blockade on the subcellular localization of SOX18 in HUVEC cells. Scale bar = 5 µm. Statistical significance was determined using ANOVA with Tukey's multiple‐comparison test. ns, non‐significance, ***P < 0.001. *n* = 3 per group. h) Effects of collagen stimulation, ITGA5‐B1 blockade, and MEK/ERK blockade on the expression of SOX18 downstream targets, MMP7 and CXCL12, in HUVEC cells. Statistical significance was determined using ANOVA with Tukey's multiple‐comparison test. **P < 0.01, ***P < 0.001. *n* = 3 per group. Abbreviations: ITGB1: integrin beta 1; ITGA5: integrin alpha 5; VWF: von‐Willebrand factor; SOX18: sex determining region Y box 18; CAF: cancer‐associated fibroblast.

To further explore the mechanisms driving collagen‐induced SOX18 upregulation, collagen receptors were investigated. The cellular effects of collagen are known to depend on its receptors.^[^ ^33^, ^34^, ^35^ ^]^ Integrin alpha 5 (ITGA5) and integrin beta 1 (ITGB1) were identified as highly expressed in endothelial cells (**Figure** **6**a,b; Figures S7 and S8, Supporting Information). In vitro assays revealed that HUVEC cells exhibited elevated levels of SOX18, ITGA5, and ITGB1 compared to tumor cells and CAFs (Figure 6c and Figure S2c, Supporting Information). In addition, collagen pre‐treatment enhanced the co‐localization of ITGA5 and ITGB1 (Figure 6d). It is well‐established that integrin α5β1, formed by ITGA5 and ITGB1,^[^ ^36^ ^]^ plays a pivotal role in promoting angiogenesis.^[^ ^37^, ^38^ ^]^ Previous studies have shown that the MEK/ERK signaling pathway is essential for SOX18 expression,^[^ ^39^ ^]^ and our in vitro assays confirmed that collagen stimulation activated the phosphorylation of MEK/ERK. Notably, inhibition of integrin α5β1 reversed the collagen‐mediated effects, downregulating SOX18 expression (Figure 6e and Figure S2d, Supporting Information). Similarly, the MAPK cascade inhibitor also blocked collagen‐induced SOX18 upregulation (Figure 6f and Figure S2e, Supporting Information). Furthermore, both integrin α5β1 and MAPK cascade inhibitors were able to block collagen‐induced nuclear translocation of SOX18 and its transcriptional activity (Figure 6g,h).In summary, the integrin α5β1/MEK/ERK signaling axis mediates collagen‐induced angiogenesis by regulating SOX18 in endothelial cells, highlighting its potential as a therapeutic target in angiogenesis.

### 2.4 LUAD Samples with Armored & Cold Phenotype Were Sensitive to Anti‐angiogenic Therapy

**Figure 7:**
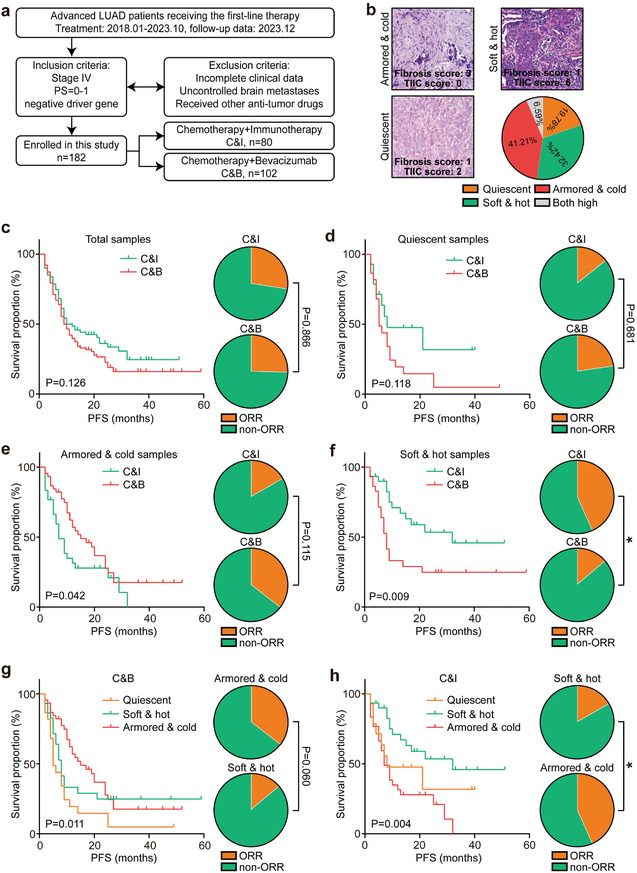
Armored & cold LUAD samples show sensitivity to anti‐angiogenic therapy. a) Overview of the processes and inclusion/exclusion criteria for selecting advanced driver gene‐negative LUAD individuals receiving first‐line therapy. b) Representative H&E images illustrating the three immuno‐collagenic subtypes in the in‐house LUAD therapy cohort. c) Therapeutic responses and prognosis of patients with LUAD receiving C&I and C&B therapies. Significance was determined using the Chi‐square test for therapeutic responses and the log‐rank test for survival analysis. *n* (C&I) = 102, *n* (C&B) = 80. d–f) Therapeutic responses and prognosis for LUAD individuals with the quiescent subtype, the armored & cold subtype, and the soft & hot subtype undergoing C&I and C&B treatments. Significance was calculated using the Chi‐square test for therapeutic responses and the log‐rank test for survival analysis. g) Therapeutic responses and prognosis for LUAD individuals with various immuno‐collagenic subtypes receiving C&B therapy. Significance was calculated using the Chi‐square test for therapeutic responses and the log‐rank test for survival analysis. h) Therapeutic responses and prognosis for LUAD individuals with various immuno‐collagenic subtypes receiving C&I therapy. Significance was calculated using the Chi‐square test for therapeutic responses and the log‐rank test for survival analysis. Abbreviations: LUAD: lung adenocarcinoma; C&I: chemotherapy plus immunotherapy; C&B: chemotherapy plus Bevacizumab; TIIC: tumor‐infiltrating immune cell; ORR: objective response rate; PFS: progression‐free survival; PS: performance status.

Previous studies have demonstrated that chemotherapy combined with immunotherapy (C&I) significantly improves prognosis in unselected LUAD populations compared to chemotherapy plus Bevacizumab (C&B). However, this benefit was not observed in certain subgroups, such as patients with PD‐L1‐negative LUAD, where the results were contradictory.^[^ ^40^, ^41^ ^]^ Based on our classification of immuno‐collagenic subtypes, PD‐L1‐negative samples were categorized as either armored & cold or quiescent subtypes, with the quiescent subtype showing no expression of angiogenesis‐related markers.^[^ ^8^ ^]^ Therefore, it was hypothesized that the armored & cold subtype would be more responsive to C&B. A retrospective study, conducted across two independent hospitals, categorized the immuno‐collagenic subtypes through HE section evaluation (**Figure** **7**a,b). The results indicated that neither unselected nor quiescent LUAD populations showed differential responses to C&I or C&B (Figure 7c,d). Notably, armored & cold tumors displayed poor responses to C&I, while soft & hot tumors showed favorable responses to this therapy (Figure 7e,f). Further subtype analysis revealed that C&B improved the prognosis of patients with armored & cold tumors, while C&I significantly enhanced the prognosis of patients with soft & hot tumors (Figure 7g,h). These results suggest that armored & cold LUAD samples, particularly those with high MVD, respond well to anti‐angiogenic therapy.

### 2.5 Correlation between Angiogenesis and Collagen Activity Was Conserved in Pan‐Cancer

**Figure 8:**
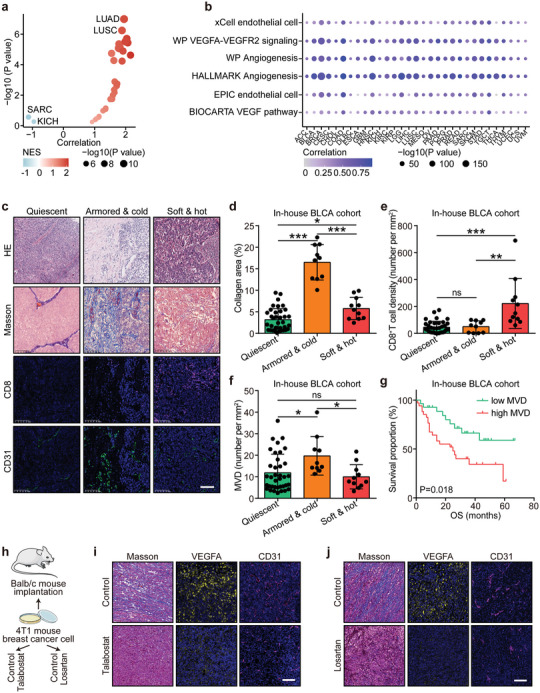
The correlation between angiogenesis and the armored & cold phenotype is conserved. a) Pan‐cancer analysis of the relationship between collagen and angiogenesis scores. Statistical significance was determined using the Pearson test. b) Correlation between collagen score and multiple angiogenesis‐related features in the TCGA pan‐cancer cohort. Significance was assessed using the Pearson test. c) Representative images showing collagen, CD8, and CD31 expression across the three immuno‐collagenic subtypes in the in‐house BLCA cohort. Scale bar = 100 µm. d–f) Quantitative analysis of collagen, CD8, and CD31 expression in the three immuno‐collagenic subtypes in the in‐house BLCA cohort. Data presented as mean ± SD. Statistical significance was calculated using the Kruskal–Wallis test with Dunn's multiple comparison for (e), and ANOVA with Tukey's multiple‐comparison test for (d) and (f). ns: not significant, *P < 0.05, **P < 0.01, ***P < 0.001. *n* (quiescent) = 37, *n* (armored & cold) = 10, *n* (soft & hot) = 11. g) Prognostic value of MVD in the in‐house LUAD cohort. Significance was determined using the log‐rank test. *n* (low) = 27, *n* (high) = 34. h) Schematic protocol of Talabostat and Losartan treatment in blab/c mice bearing 4T1 cells. Paraffin‐embedded tumor samples from the above models and the two mouse tumor models with collagen inhibition were submitted for CD31 and VEGFA immunofluorescence staining and Masson staining. i,j) Collagen deposition levels and expression of VEGFA and CD31 in mouse tumor tissues. Scale bar = 100 µm. Abbreviations: TCGA: The Cancer Genome Atlas; BLCA: bladder cancer; LUAD: lung adenocarcinoma; MVD: microvessel density; VEGFA: vascular endothelial growth factor A; SARC: sarcoma; KICH: kidney chromophobe; LUSC: lung squamous carcinoma.

Given the consistency of immuno‐collagenic subtypes across cancers, the correlation between angiogenesis and collagen activity was explored in pan‐cancer. Analysis of the TCGA dataset revealed a positive correlation between collagen and angiogenesis scores in most solid tumor types, with the exception of sarcoma (SARC) and kidney chromophobe (KICH) (**Figure** **8**a). Additionally, the collagen score showed a strong correlation with six angiogenesis‐related activities (Figure 8b). Given the strong prognostic and immune response predictive value in bladder cancer (BLCA),^[^ ^8^ ^]^ these findings were validated in BLCA samples. Similar to the results from the in‐house LUAD cohort, armored & cold tumors in BLCA exhibited high collagen expression and low CD8 expression (Figure 8c–e). Notably, the MVD was highest in armored & cold tumors (Figures 8c and 7f), and elevated MVD correlated with poor prognosis in the BLCA cohort (Figure 8g). Further analysis confirmed a positive correlation between increased collagen levels and MVD in BLCA samples (Figure S9a,b, Supporting Information). In summary, the correlation between angiogenesis and collagen activity is conserved across pan‐cancer, suggesting that cancer types with an armored & cold phenotype are particularly suited for anti‐angiogenic therapies.

### 2.6 Armored & Cold Evolution Evoked by Immunotherapy Reveals Anti‐angiogenic Therapy

**Figure 9:**
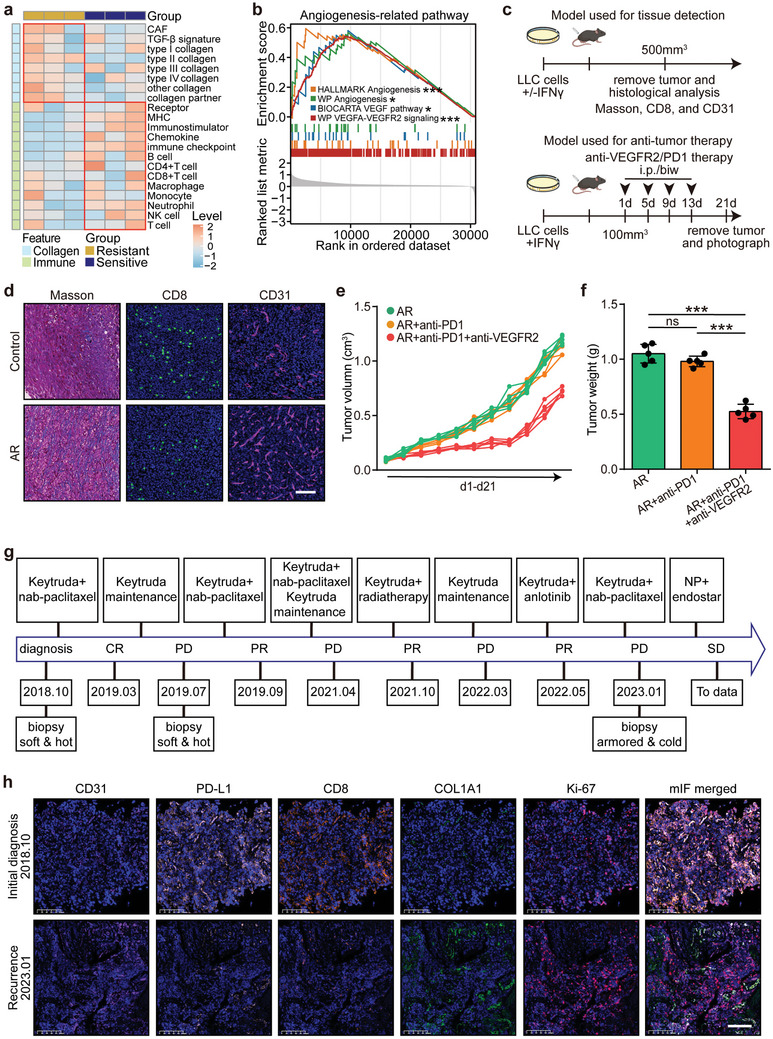
Acquired resistance to immunotherapy mediated by armored & cold evolution highlights the potential for anti‐angiogenic therapy. a) Heatmap displaying collagen‐ and immune‐related features in sensitive versus acquired resistant tissues from the GSE164357 dataset. b) GSEA analysis of the correlation between acquired resistance‐related genes and four angiogenesis‐related features, including “HALLMARK Angiogenesis,” “WP Angiogenesis,” “BIOCARTA VEGF pathway,” and “WP VEGFA‐VEGFR2 signaling.” c) Schematic of the establishment of an acquired resistance mouse model and anti‐VEGFR2 treatment in C57BL/6 mice bearing Lewis cells. d) Collagen deposition levels and expression of T cell marker CD8 and endothelial marker CD31 in mouse tumor tissues. Original magnification: 200×. e,f) Effects of anti‐PD‐1 and anti‐VEGFR2 treatment on tumor volume and weight in C57BL/6 mice bearing Lewis cells. Data presented as mean ± SD. Statistical significance determined using Student's *t*‐test. ns: not significant, ***P < 0.001. *n* = 5 per group. g) Treatment timeline for a patient who underwent immunotherapy, combined with chemotherapy, radiotherapy, and Anlotinib. After four progressions, clinical benefits were again observed with Vinorelbine + Platinum (NP) and Endostar. h) Multiplex immunohistochemistry staining (CD31, CD8, PD‐L1, COL1A1, and Ki67) of tumor samples at initial diagnosis and fourth progression. Scale bar = 100 µm. Abbreviations: VEGFA: vascular endothelial growth factor A; VEGFR2: vascular endothelial growth factor receptor 2; NP: Vinorelbine + Platinum; AR: acquired resistance; PD: progression disease; SD: stable disease; PR: partial response; CR: complete response.

Immunotherapy can elicit profound and durable responses in certain cancer individuals. However, more than half of those who initially respond eventually develop progression, termed acquired resistance.^[^ ^42^ ^]^ The underlying mechanisms of this resistance remain poorly understood. Tumor evolution toward an armored & cold phenotype may play a critical role in acquired immunotherapy resistance. Analysis of the GSE164357 dataset revealed that tumor tissues with acquired resistance exhibited increased collagen signatures and reduced immune signatures (**Figure** **9**a). In addition, acquired resistance correlated positively with angiogenesis‐related pathways, including the HALLMARK angiogenesis signature, WP angiogenesis signature, BIOCARTA VEGF pathway, and WP VEGFA‐VEGFR2 signaling (Figure 9b). A mouse model of acquired immunotherapy resistance, established as previously described^[^ ^43^ ^]^ (Figure 9c), showed that tumors with acquired resistance had increased collagen deposition and vascular endothelial cell infiltration, alongside decreased T cell infiltration (Figure 9d). As anticipated, anti‐PD‐1 therapy failed to inhibit tumor growth, while anti‐VEGFR2 therapy effectively restored the sensitivity to the anti‐PD‐1 therapy in this model (Figure 9e,f). These results indicate that while armored & cold evolution is not the sole factor, it is a significant contributor to acquired resistance to immunotherapy. In a previous case report, a patient with cancer of unknown primary (CUP) initially benefited from immunotherapy but eventually progressed.^[^ ^44^ ^]^ Remarkably, cancer progression was controlled by anti‐angiogenic therapy with Endostatin (Figure 9g). Molecular analysis of tumor samples from the initial diagnosis and recurrence revealed that the tumor evolved to display an armored & cold phenotype, with an increased proliferation index (Figure 9h). In conclusion, tumor evolution to the armored & cold phenotype significantly contributes to acquired resistance to immunotherapy, suggesting that anti‐angiogenic therapy may offer potential therapeutic benefits.

## 3 Discussion

Collagen, a key component of the ECM, is aberrantly deposited within the TME, surrounding tumor cell clusters. This deposition has several detrimental effects: 1) it obstructs direct interactions between immune cells and tumor cells;^[^ ^45^ ^]^ 2) it physically restricts the cytotoxicity of immune cells;^[^ ^46^ ^]^ and 3) it releases bioactive fragments that modulate immune cell activity.^[^ ^47^ ^]^ Previous work proposed a framework that integrates collagen characteristics with immune signatures to predict responses to ICB therapy. Among the identified subtypes, armored & cold tumors exhibited the most favorable response to ICB across various cancer types.^[^ ^8^ ^]^ In addition, an unbiased approach highlighted potential drug targets, primarily stromal proteins such as B7‐H3 and vascularization markers, which are highly expressed in armored & cold tumors and could be leveraged for targeted therapy.^[^ ^8^ ^]^

Angiogenesis is a critical driver of tumor progression across diverse cancer types,^[^ ^48^, ^49^, ^50^ ^]^ regulated by a complex network of factors. Pro‐angiogenic molecules, including VEGF,^[^ ^51^ ^]^ IL‐8,^[^ ^52^ ^]^ TGF‐β,^[^ ^53^ ^]^ and PDGF,^[^ ^54^ ^]^ promote angiogenesis and facilitate cancer metastasis. Furthermore, various TME cells, such as cancer‐associated fibroblasts,^[^ ^6^ ^]^ mast cells,^[^ ^55^ ^]^ and tumor‐associated macrophages,^[^ ^56^, ^57^ ^]^ play essential roles in regulating angiogenesis and tumor progression. In our research, armored & cold tumors exhibited the highest levels of angiogenesis activity in multiple cancer types. These tumors were characterized by low immune cell infiltration and high collagen deposition.^[^ ^8^ ^]^ The elevated collagen levels in the TME contributed to enhanced angiogenic activity. Mechanistically, collagen promoted VEGFA expression in tumor cells and directly facilitated vessel formation and the proliferation of HUVEC endothelial cells by upregulating SOX18 expression and its transcriptional activity.

Anti‐angiogenic therapy has become an established strategy in oncology.^[^ ^58^ ^]^ The primary objective of anti‐angiogenic therapy is to transform cancer into a “dormant” state by disrupting the tumor's blood supply, effectively “starving” tumor cells.^[^ ^59^ ^]^ Similar to immunotherapy, anti‐angiogenic therapies offer broad anti‐tumor activity, demonstrating significant efficacy in cancers such as lung cancer,^[^ ^60^ ^]^ hepatocellular carcinoma,^[^ ^61^ ^]^ and colorectal cancer.^[^ ^62^ ^]^ However, unlike immunotherapy, there is currently no stable or reliable biomarker to identify patients who would benefit from anti‐angiogenic therapy. Nonetheless, several clinical trials have shown that high MVD correlates with prolonged survival in patients treated with anti‐angiogenic agents, particularly Bevacizumab.^[^ ^20^, ^63^, ^64^ ^]^

To assess the impact of immuno‐collagenic subtypes on therapeutic decision‐making, an advanced LUAD cohort was selected for validation. LUAD, one of the most prevalent and aggressive cancers, is typically treated with standard regimens such as C&I or C&B.^[^ ^65^, ^66^, ^67^, ^68^ ^]^ Both treatments significantly prolong survival in advanced stages of LUAD.^[^ ^69^, ^70^ ^]^ However, therapeutic responses to these regimens vary widely among patients. As observed in public cohorts, armored & cold tumors are more responsive to first‐line C&B therapy. Moreover, emerging combination strategies involving chemotherapy, immunotherapy, and anti‐angiogenic therapy^[^ ^71^ ^]^ show promise in improving the outcomes of patients with LUAD. Despite these advancements, the economic burden and side effects associated with quadruple therapy make it unnecessary for patients with armored & cold tumors. Given the immune‐activating properties of anti‐angiogenic therapy,^[^ ^72^, ^73^ ^]^ a more feasible approach might be sequential immunotherapy following anti‐angiogenic therapy for patients with armored & cold tumors.

It has been shown that anti‐tumor therapies can alter the TME to some extent.^[^ ^74^, ^75^ ^]^ One of the proposed mechanisms of acquired resistance to immunotherapy is the increased fibrotic signature within the TME.^[^ ^35^, ^76^, ^77^ ^]^ This hypothesis was further supported by analyses of the GSE164357 dataset, which revealed that tumors with acquired immunotherapy resistance exhibited an increased fibrotic signature, alongside a decrease in immune activity—phenotypically shifting to an armored & cold profile. In addition, a previously reported case of CUP initially responded well to immune checkpoint blockade but eventually progressed into an armored & cold tumor with high MVD levels after prolonged immunotherapy.^[^ ^44^ ^]^ Encouragingly, despite the transformation to an armored & cold phenotype, anti‐angiogenic therapy plus anti‐PD‐1 therapy effectively inhibited tumor growth. Moreover, evidences from clinical trials also supported our academic point. The VARGADO study uncovered that anti‐angiogenic Nintedanib plus Docetaxel represented an efficient treatment option after failure of prior immunotherapy in non‐small cell lung cancer.^[^ ^78^ ^]^ The Lung‐MAP S1800A study suggested that anti‐angiogenic Ramucirumab and Pembrolizumab improved the OS in advanced non‐small cell lung cancer previously treated with immunotherapy.^[^ ^79^ ^]^ Thus, even in the context of treatment‐induced armored & cold tumor evolution, anti‐angiogenic therapy remains a potentially effective therapeutic strategy.

## 4 Conclusions 

This study demonstrates that armored & cold tumors consistently exhibit elevated MVD levels across various cancer types. Mechanistically, collagen fosters angiogenesis by directly activating endothelial cells and increasing the expression and secretion of VEGFA in tumor cells. Clinically, for refractory armored & cold tumors, C&B therapy offers more significant benefits compared to C&I. To sum up, we reported that collagen deposition promoted angiogenesis in various cancers, and offered a novel and robust perspective for the challenging decision‐making process regarding the selection of chemotherapy plus anti‐angiogenic therapy. However, clinical data from other cancer types have not been collected to validate the conclusions drawn in LUAD. Thus, the applicability of clinical conclusions to cancer types other than LUAD requires independent validation.

## 5 Experimental Section

### Transcriptome Data from Public Portals

Transcriptome datasets and clinical annotations for 9363 cancer individuals across 31 solid tumor types were retrieved from the TCGA dataset via the University of California Santa Cruz (UCSC) Xena platform (https://xenabrowser.net/datapages/). Only samples with complete clinical data were selected for further analysis. Single‐cell RNA sequencing (scRNA‐seq) data from 13 LUAD samples were obtained from the GSE131907^[^ ^80^ ^]^ and GSE149655^[^ ^81^ ^]^ datasets, while spatial transcriptomics data from 12 LUAD samples were sourced from the E‐MTAB‐13530 dataset.^[^ ^26^ ^]^ Gene expression profiles of tumor samples with varying sensitivities to immunotherapy were retrieved from the GSE164357 dataset.^[^ ^82^ ^]^

### ScRNA‐seq and Spatial Transcriptomics Data Analysis

For scRNA‐seq data processing, cells were excluded if mitochondrial gene expression exceeded 10%, or if fewer than 200 or more than 5000 genes were detected. A total of 46108 cells were analyzed. To minimize batch effects across the 13 samples, the “RunHarmony” function^[^ ^83^ ^]^ was applied. Principal component analysis (PCA) was conducted on the top 4000 variable genes, utilizing the first 40 principal components (PCs) to reduce dimensionality. Cells were annotated into eight cell types based on conventional markers, such as DCN for fibroblasts and VWF for endothelial cells. Cell–cell interactions were assessed using the “CellPhoneDB” software,^[^ ^84^ ^]^ which identifies significant ligand–receptor pairs (*P* < 0.05) to characterize communications between different cell types.

For spatial transcriptomics data, a total of 29185 spots were collected and normalized using log‐normalization.^[^ ^85^ ^]^ Data were processed similarly to scRNA‐seq data to eliminate batch effects and perform unsupervised clustering. Six cell types were annotated based on established markers, and Euclidean distance was used to calculate the spatial proximity between cell pairs based on their coordinates.

### Enrichment Analysis

To explore the biological processes in patients with the armored & cold phenotype, differential expression analysis was performed using the “limma” package.^[^ ^86^ ^]^ Genes with a *P*‐value < 0.05 and fold change (FC) ≥ 1.5 were classified as armored & cold‐related genes. Enrichment analysis was conducted using the “GSEA” function in the “clusterProfiler” package^[^ ^87^ ^]^ with “HALLMARK” gene sets,^[^ ^88^ ^]^ and angiogenesis‐related pathways were retrieved from the GSEA database (https://www.gsea‐msigdb.org).

### Definition of Angiogenesis Signature

Gene markers for various angiogenesis signatures were collected from relevant studies (Table S1, Supporting Information). The enrichment scores of these signatures were calculated using the Gene Set Variation Analysis (GSVA) R package.^[^ ^89^ ^]^ Angiogenesis activity was assessed based on the average values of these angiogenesis signatures.

### Clinical Samples

Two paraffin‐embedded tumor microarrays (TMAs) (catalogs HBlaU079Su01 and HLugAde150Su02) were obtained from the National Engineering Center for Biochip (Outdo Biotech, Shanghai, China), with approval from the Outdo Biotech Clinical Research Ethics Committee (No. SHYJS‐CP‐1910002 and SHYJS‐CP‐1404016).

To evaluate the impact of immuno‐collagenic subtypes on treatment decisions, a real‐world study involving advanced, driver gene‐negative LUAD individuals receiving first‐line treatment was conducted. To minimize the influence of chemotherapy on immunotherapy and anti‐angiogenic therapy, the ESMO and CSCO guidelines were followed,^[^ ^90^ ^]^ selecting pemetrexed plus platinum as the background chemotherapy. The study included two treatment arms: chemotherapy plus immunotherapy (C&I arm), consisting of pemetrexed, a platinum‐based drug, and a PD‐1 monoclonal antibody; and chemotherapy plus anti‐angiogenic therapy (C&B arm), consisting of pemetrexed, a platinum‐based drug, and Bevacizumab. A total of 182 patients were recruited from The First Affiliated Hospital of Nanjing Medical University and The Affiliated Wuxi People's Hospital of Nanjing Medical University between January 2018 and October 2023, with ethical approval (No. 2024‐SR‐017 and KY23176). Efficacy was assessed using the RECIST 1.1 criteria, measuring progression‐free survival (PFS), with a follow‐up cutoff of December 2023. No significant differences were observed in baseline features between the cohorts, except for gender (Table S2, Supporting Information). In addition, biopsy samples from initial diagnosis and relapse following ineffective immunotherapy from a previously reported CUP case^[^ ^44^ ^]^ at The Affiliated Wuxi People's Hospital of Nanjing Medical University were collected for immunofluorescence and Masson staining, with ethical approval (No. KY23176).

### Cell Lines and Cell Culture

Human cancer cell lines A549 (catalog KGG3215‐1) and NCI‐H1299 (catalog KGG3216‐1) were purchased from KeyGEN (Nanjing, China). The vascular endothelial cell line HUVEC (catalog SC0396) was purchased from YUCHI Biology (Nanjing, China). A549 cells were maintained in an F12K medium supplemented with 10% fetal bovine serum (FBS) at 37 °C with 5% CO_2_. NCI‐H1299 cells were cultured in RPMI‐1640 medium supplemented with 10% FBS at 37 °C with 5% CO_2_. HUVEC cells were cultured in endothelial cell medium (catalog 1001, ScienceCell, California, USA) at 37 °C with 5% CO_2_. CAFs were extracted as previously described.^[^ ^91^ ^]^ All experiments were performed using mycoplasma‐free cells, and these cell lines were recently authenticated via short tandem repeat profiling.

### In Vitro Inhibition of SOX18, Integrin α5β1, and MAPK Cascade

The SOX18 inhibitor, Sm4,^[^ ^92^ ^]^ was purchased from MedChemExpress (Catalog HY‐116940), with a working concentration of 10 µmol L^−1^ for in vitro assays. The integrin α5β1 inhibitor, ATN‐161,^[^ ^93^ ^]^ was obtained from TargetMol (catalog T10397) at a working concentration of 1 µmol L^−1^, and the MAPK inhibitor, U0126,^[^ ^94^ ^]^ was purchased from TargetMol (catalog T21332) with a working concentration of 5 µmol L^−1^ for in vitro assays.

### Collagen Coating

For cell culture, type I collagen (catalog A1048301, Gibco, Thermo Fisher Scientific, MA, USA) was applied to culture plates following the manufacturer's instructions. Collagen was diluted to 0.1 mg mL^−1^ in sterile 0.006 mol L^−1^ acetic acid, and 60% of the medium was added to the diluted collagen. This mixture was allowed to dry for 1 h at room temperature under a sterile hood. The final concentration used in vivo assays was 10 µg cm^−2^.

### Tube Formation Assay In Vitro

For the tube formation assay, 50 µL of diluted Matrigel (catalog KGL5101, KeyGEN, Nanjing, China) was added to each well of a 96‐well plate and allowed to solidify for 2 h at 37 °C. HUVECs, with or without collagen stimulation for 24 h, were seeded onto the Matrigel‐coated wells (3 × 10^4^ cells in 150 µL) and incubated for 6 h at 37 °C. Tube‐like structures were quantified by counting from three random microscopic fields at 100× magnification.

### Cell Proliferation, Migration, and Apoptosis Assays

Cell proliferation of HUVECs was assessed using the CCK‐8 assay. Briefly, suspended HUVECs were seeded onto a 96‐well plate, with or without collagen coating, at a density of 5 × 10^3^ cells mL^−1^ (100 µL/well) and incubated at 37 °C with 5% CO_2_ for 24 h. CCK‐8 reagent (10 µL, catalog KGA9310, KeyGEN, Nanjing, China) was then added to each well, and the plate was incubated for 1 h. The optical density (OD) at 450 nm was measured using a microplate reader. Cell migration was assessed using Transwell chambers without Matrigel coating. HUVECs, with or without collagen stimulation for 24 h, were digested with 0.25% trypsin for 1 min and resuspended. A total of 5 × 10^4^ HUVECs in 200 µL serum‐free medium were seeded in the upper chamber, while 600 µL of medium with 10% FBS was added to the lower chamber. After 24 h, migrated cells on the lower side of the membrane were fixed with 4% paraformaldehyde and stained with 0.2% crystal violet. Migrated cells were quantified by counting stained cells from three random microscopic fields at 100× magnification. Cell apoptosis in treated HUVECs was analyzed using the Annexin V‐FITC/PI Kit (Catalog KGA1102, KeyGEN) according to the manufacturer's instructions. Since necrotic cells and late apoptotic cells cannot be distinguished by Annexin V and PI staining, the manufacturer recommends calculating only the early apoptotic cells.

### RNA‐Sequencing Analysis

For the RNA‐sequencing (RNA‐seq) assay, total RNA from HUVEC cells, with or without collagen stimulation, was submitted to TENK Genomics for processing. Gene expression levels were quantified using the Fragments Per Kilobase of Exon Model per Million Mapped Fragments (FPKM). GSEA was then performed to assess the association between collagen‐induced gene expression changes and angiogenesis‐related pathways. Differentially expressed genes were defined as those with a *P*‐value < 0.05 and an FC ≥ 1.5.

### Western Blotting Analysis and Enzyme‐Linked Immunosorbent Assay (ELISA)

For protein analysis, human cancer cells were plated in six‐well plates for collagen stimulation. After 48 hours, total protein from A549 and NCI‐H1299 cells was harvested using a lysis buffer. SDS‐PAGE and Western blotting were performed according to standard protocols. The primary antibodies used for Western blotting included: VEGFA (1:1000 dilution, catalog ab214424, Abcam), SOX18 (1:1000 dilution, catalog A04004‐2, Boster), ITGA5 (1:1000 dilution, catalog A19069, Abclonal, Wuhan, China), ITGB1 (1:1000 dilution, catalog A23497, Abclonal), p‐MEK (1:1000 dilution, catalog 9154, Cell Signaling Technology), MEK (1:1000 dilution, catalog A4868, Abclonal), p‐ERK (1:1000 dilution, catalog 9102, Cell Signaling Technology), ERK (1:1000 dilution, catalog A4782, Abclonal), Vinculin (1:5000 dilution, catalog 66305‐1‐Ig, ProteinTech, Wuhan, China), and GAPDH (1:5000 dilution, catalog 60004‐1‐Ig, ProteinTech). In addition, soluble VEGFA levels in the cancer cell medium were measured using an ELISA assay. The VEGFA ELISA kit (catalog E‐EL‐H0111) was obtained from Elabscience (Wuhan, China), and all samples were analyzed in duplicates according to the manufacturer's instructions.

### Quantitative Real‐Time PCR

Total RNA was extracted from cells using Trizol reagent (catalog KGF5101, KeyGEN, Nanjing, China). Primers for MMP7, CXCL12, and GAPDH mRNA reverse transcription were synthesized by KeyGEN (Nanjing, China). qRT‐PCR was conducted using the One‐Step TB Green PrimeScript RT‐PCR Kit II (SYBR Green) (catalog RR086B, TaKaRa, Kyoto, Japan). The primers used for amplification were as follows:

MMP7: (forward) 5′‐CTACAGTGGGAACAGGCTCAG‐3′, (reverse) 5′‐TGCATCTCCTTGAGTTTGGCT‐3′

CXCL12: (forward) 5′‐TACAGATGCCCATGCCGATTC‐3′, (reverse) 5′‐GGCACAGTTTGGAGTGTTGAG‐3′

GAPDH: (forward) 5′‐AGATCATCAGCAATGCCTCCT‐3′, (reverse) 5′‐TGAGTCCTTCCACGATACCAA‐3′.

### Immunofluorescence

The subcellular locations of SOX18 were assessed using immunofluorescence assay according to standardized protocols. The primary antibodies utilized were as follows: ITGA5 (1:200 dilution, catalog sc16665, Santa Cruz Biotechnology), ITGB1 (1: 200 dilution, catalog A23497, Abclonal), and SOX18 (1: 200 dilution, catalog A04004‐2, Boster). The stained cells were visualized using a fluorescence microscope.

### Tumor‐Bearing Mouse Model and Drug Treatment

C57BL/6 mice (4–5 weeks old) were purchased from Suzhou SPFbiotech Co., Ltd. The mice were raised in SPF‐grade experimental animal centers and provided with free access to food and water. To establish the syngeneic mouse model, Lewis mouse lung tumor cells (catalog KGG2202‐1, KeyGEN, Nanjing, China) maintained in DMEM media supplemented with 10% FBS were subcutaneously injected into the flanks of these male mice (5 × 10^6^ cells). Tumors were monitored and regularly measured with calipers every two to three days. Upon tumors reaching an average size of ≈100 mm^3^, tumor‐bearing mice were randomly assigned to groups. The control group received oral administration of phosphate balanced solution (PBS). The talabostat group received daily oral administration of talabostat (catalog HY‐13233A, MedChemExpress) at 20 µg/mouse. The tumors were removed from the unconscious animals at day 21 after the initiation of the treatment, which was subsequently documented and weighed. Paraffin‐embedded tumor samples from the above models and the two mouse tumor models with collagen inhibition^[^ ^8^, ^91^ ^]^ were submitted for CD31 and VEGFA immunofluorescence staining and Masson staining.

In addition, to test the therapeutic effects of anti‐VEGFR2 therapy on mouse with acquired resistant tumors to immunotherapy, we established mouse models with acquired resistance to immunotherapy according to previous study.^[^ ^43^ ^]^ Paraffin‐embedded tumor samples from the models with acquired resistance to immunotherapy and control models were submitted for CD31 and CD8 immunofluorescence staining and Masson staining. Then, anti‐PD‐1 therapy and anti‐VEGFR2 therapy were conducted in the models with acquired resistance to immunotherapy. Anti‐PD‐1 therapy was intraperitoneally injected with anti‐mouse PD‐1 antibody (catalog BE0273, clone 29F.1A12, BioXCell) at 200 µg/mouse twice a week. Anti‐VEGFR2 therapy was intraperitoneally injected with anti‐mouse VEGFR2 antibody (catalog A2126, clone DC101, Selleck) at 100 µg/mouse twice a week. All animal experiments were approved by the Laboratory Animal Ethics Committee at Nanjing Medical University (No. IACUC‐2312041)

### Multiplexed Immunohistochemistry and Histochemistry Staining

Paraffin‐embedded TMAs were submitted for multiplexed immunohistochemistry (mIHC), hematoxylin and eosin (HE), and Masson staining. Standard operating procedures were employed for mIHC, HE, and Masson staining. The primary antibodies used for immunofluorescence staining were as follows: anti‐CD8 (Ready‐to‐use, catalog AF20211, AiFang Biology, Changsha, China), anti‐CD31 (Ready‐to‐use, catalog AF20112, AiFang Biology, Changsha, China), anti‐PD‐L1 (Ready‐to‐use, catalog AF20084, AiFang Biology, Changsha, China), anti‐COL1A1 (Ready‐to‐use, catalog AF20400, AiFang Biology, Changsha, China), and anti‐Ki67 (Ready‐to‐use, catalog AF20068, AiFang Biology, Changsha, China). Masson's staining was conducted using the Trichrome Stain kit (catalog FH115100, FreeThinking, Nanjing, China) following the manufacturer's instructions. For HE staining, the TIIC score was assessed using the criteria established by the TCGA Network^[^ ^95^ ^]^ and the fibrosis score was assessed according to a previous study^[^ ^96^ ^]^ by two senior pathologists. Tumor samples were categorized based on TIIC score and fibrosis score: soft & hot tumors (TIIC score ≥ 3 and fibrosis score 0‐1), armored & cold tumors (TIIC score ≤ 2 and fibrosis score 2‐3), and quiescent tumors (TIIC score ≤ 2 and fibrosis score 0‐1). In addition, immunofluorescence staining was evaluated using HALO software to determine the number of CD8 positive cells, and the MVD was counted by two senior pathologists. Masson staining was evaluated using HALO software to determine positive‐stained area percentages.

### Statistical Analysis

Statistical analyses and figure presentations were conducted using R language 4.0.2 and GraphPad Prism 6.0. Group differences were assessed using the Student's *t*‐test or Mann–Whitney test for two groups, while one‐way analysis of variance or the Kruskal–Wallis test with multiple comparisons was utilized for multiple groups. Categorical variables were assessed using the chi‐square test or Fisher exact probability test. Pearson's or Spearman's correlation tests were used to evaluate correlations between variables. Receiver‐operating characteristic (ROC) curve analysis was utilized to determine the specificity and sensitivity of candidate indicators, generating the area under the ROC curve for diagnostic biomarkers. Prognostic values of categorical variables were assessed via the log‐rank test. A *P*‐value of <0.05 was considered statistically significant for all analyses.

## Conflict of Interest

The authors declare no conflict of interest.

## Author Contributions

J.M., K.Y., X.Z., and Z.L. contributed equally to this work. Y.Y., Y.C., and J.X. conceived the study and participated in the study design, performance, coordination, and manuscript writing. J.M., X.Z., Z.L., J.C., and Y.Z. carried out the in vivo and in vitro assays. Y.C. and J.M. conducted bioinformatics analysis. K.Y., M.T., H.F., J.D., and J.X. collected the clinical cohorts. Y.Y. and Y.C. revised the manuscript. Y.Y. and J.X. got the financial support. All authors reviewed and approved the final manuscript.

## Availability of Data and Materials

All data generated or analyzed during this study are included in this published article and its additional files.

## Ethics Approval and Consent to Participate

Ethical approval for the use of TMAs was granted by the Clinical Research Ethics Committee in Outdo Biotech (No. SHYJS‐CP‐1910002 and SHYJS‐CP‐1404016). Ethical approval for the collection of the clinical cohort from The Affiliated Wuxi People's Hospital of Nanjing Medical University was granted by the Clinical Research Ethics Committee in The Affiliated Wuxi People's Hospital of Nanjing Medical University (No. KY23176). Ethical approval for the collection of the clinical cohort from The First Affiliated Hospital of Nanjing Medical University was granted by the Clinical Research Ethics Committee in The First Affiliated Hospital of Nanjing Medical University (No. 2024‐SR‐017). All animal experiments were approved by the Laboratory Animal Ethics Committee at Nanjing Medical University (No. IACUC‐2312041).

## Consent for Publication

All authors agreed on the manuscript.

## Supporting information



Supporting Information

## Data Availability

The data that support the findings of this study are available from the corresponding author upon reasonable request.
