# Supplementary material for: Intratumoral Collagen Deposition Supports Angiogenesis Suggesting Anti‐angiogenic Therapy in Armored and Cold Tumors
Source: Adv Sci (Weinh). 2025 Jan 17;12(10):2409147. doi: 10.1002/advs.202409147 (PMC11904994; doi:10.1002/advs.202409147)
Supplement: Supplementary file 1 — Supporting Information [file ADVS-12-2409147-s001.docx]

**
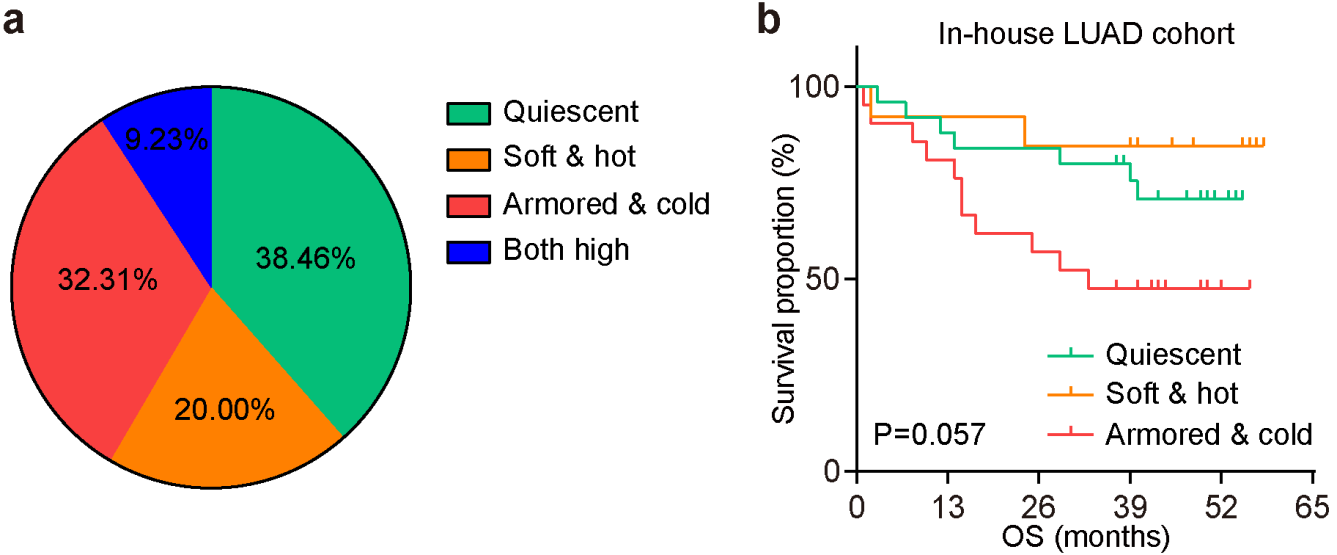
**

**Figure S1. Subtyping of the in-house LUAD cohort.**

(a) Proportion of four subtypes divided by fibrosis score and TIIC infiltration. The proportion of co-high fibrosis and TIIC was limited as shown on the pie chart. (b) Difference in overall survival in three immuno-collagenic subtypes in the in-house LUAD cohort. Significance was calculated using the log-rank test.


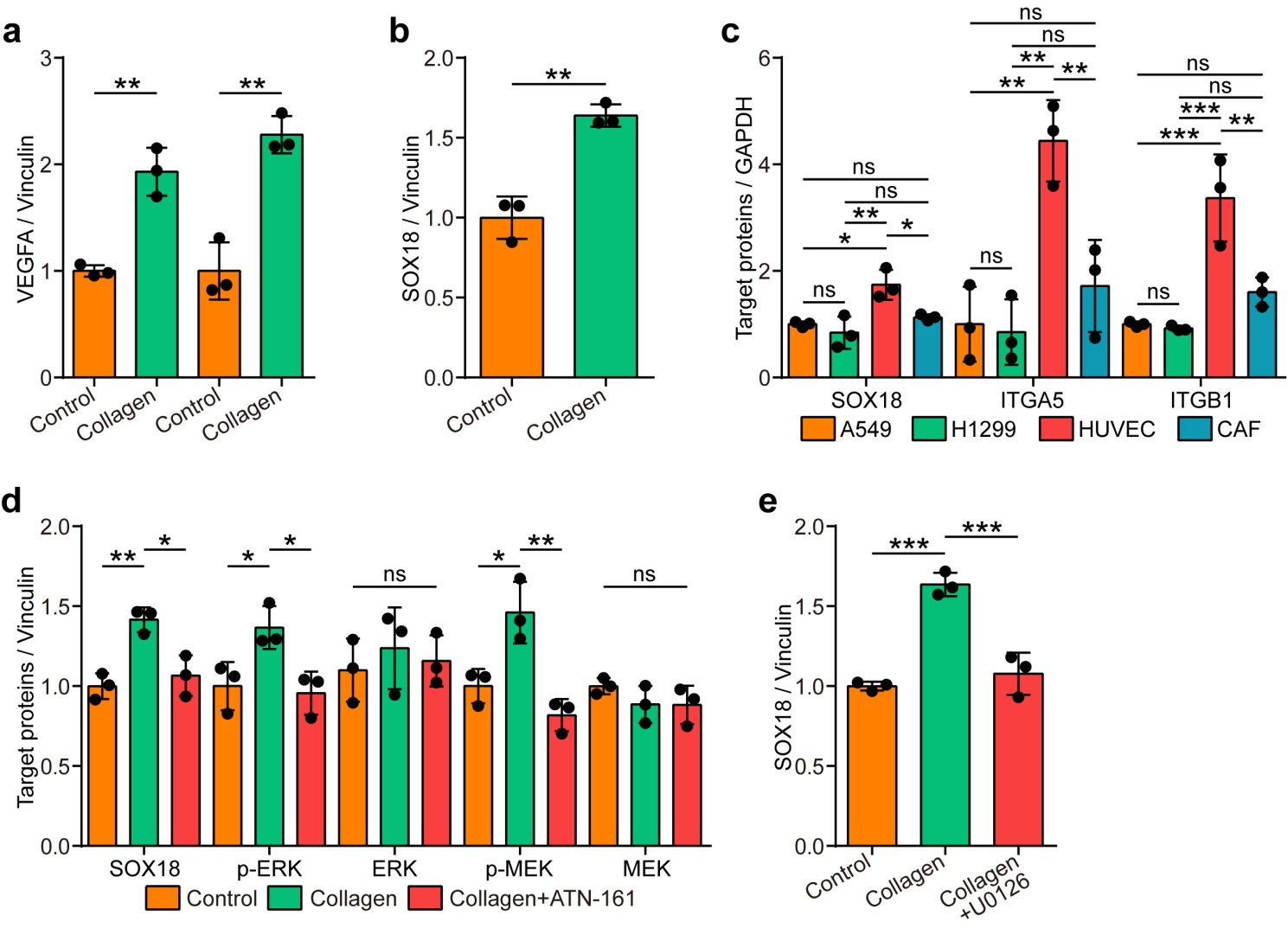


**Figure S2. Quantitative analysis of Western blotting assays.** (a) Figure 3i. (b) Figure 5e. (c) Figure 6c. (d) Figure 6e. (e) Figure 6f. n =3 per group.

**
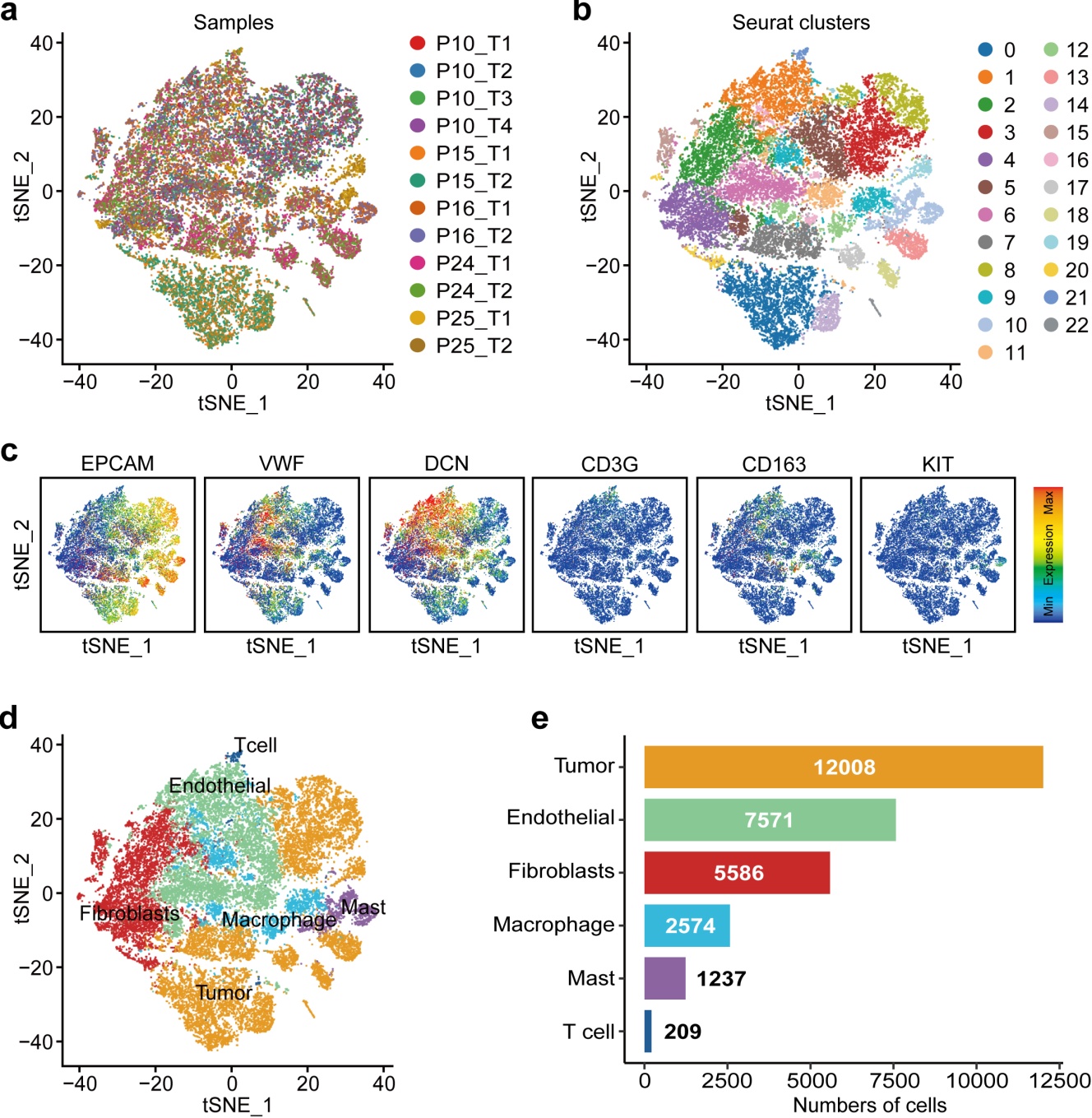
**

**Figure S3. Unsupervised clustering and cell type annotation of spatial transcriptomics data from twenty LUAD tumor tissues.** (a) t-SNE visualization of 29,185 single cells passed quality controls, colored by 12 LUAD samples. (b) The unsupervised clustering of 29,185 cells. (c) Expression levels of cell-type-specific genes overlaid on the t-SNE representation. (d) t-SNE visualization of cell types annotated by established signatures. (e) Bar plot showing the number of cells for each cell type.

**
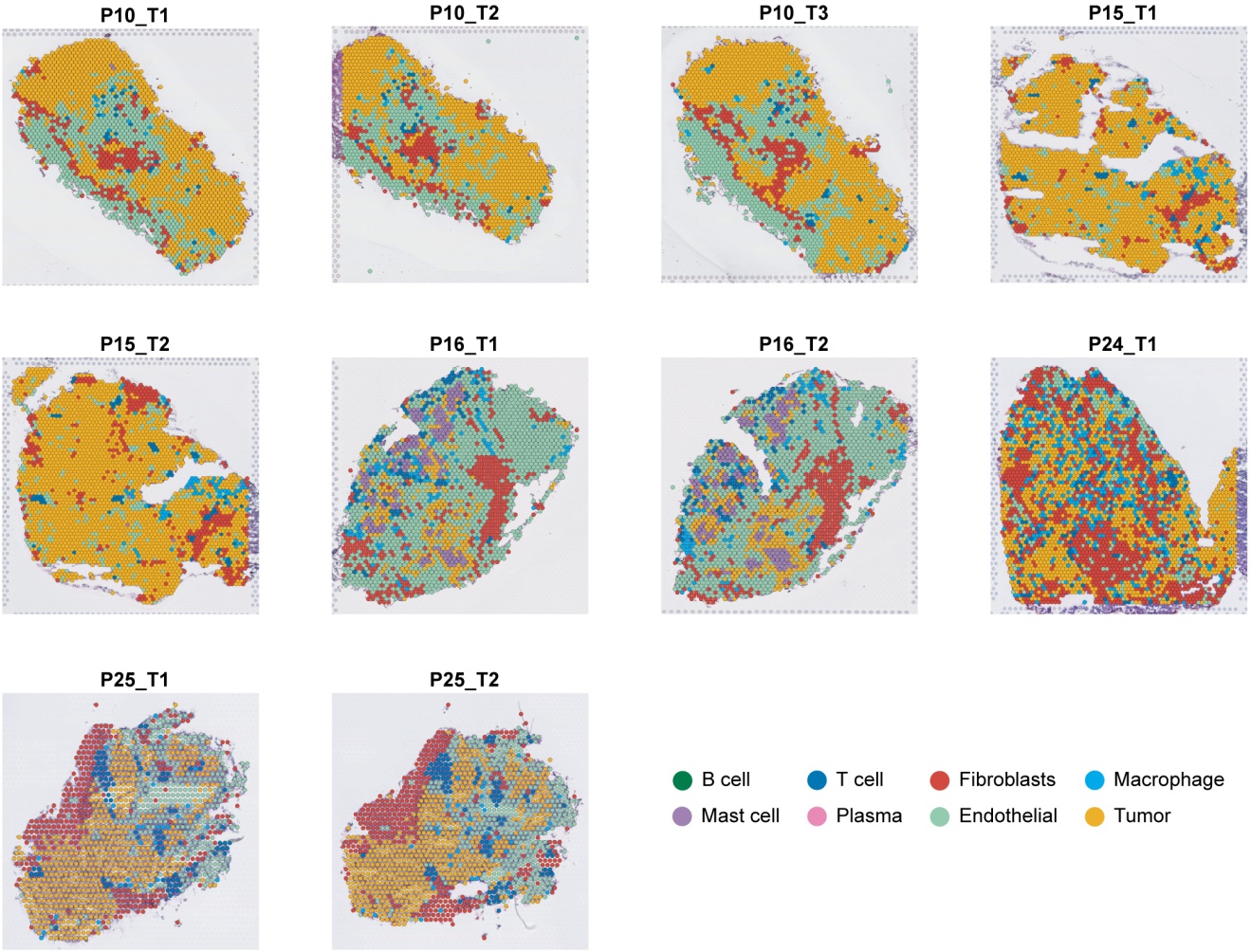
**

**Figure S4. Spatial cell charting of LUAD samples.** A total of 10 samples from 5 LUAD tumors.

**
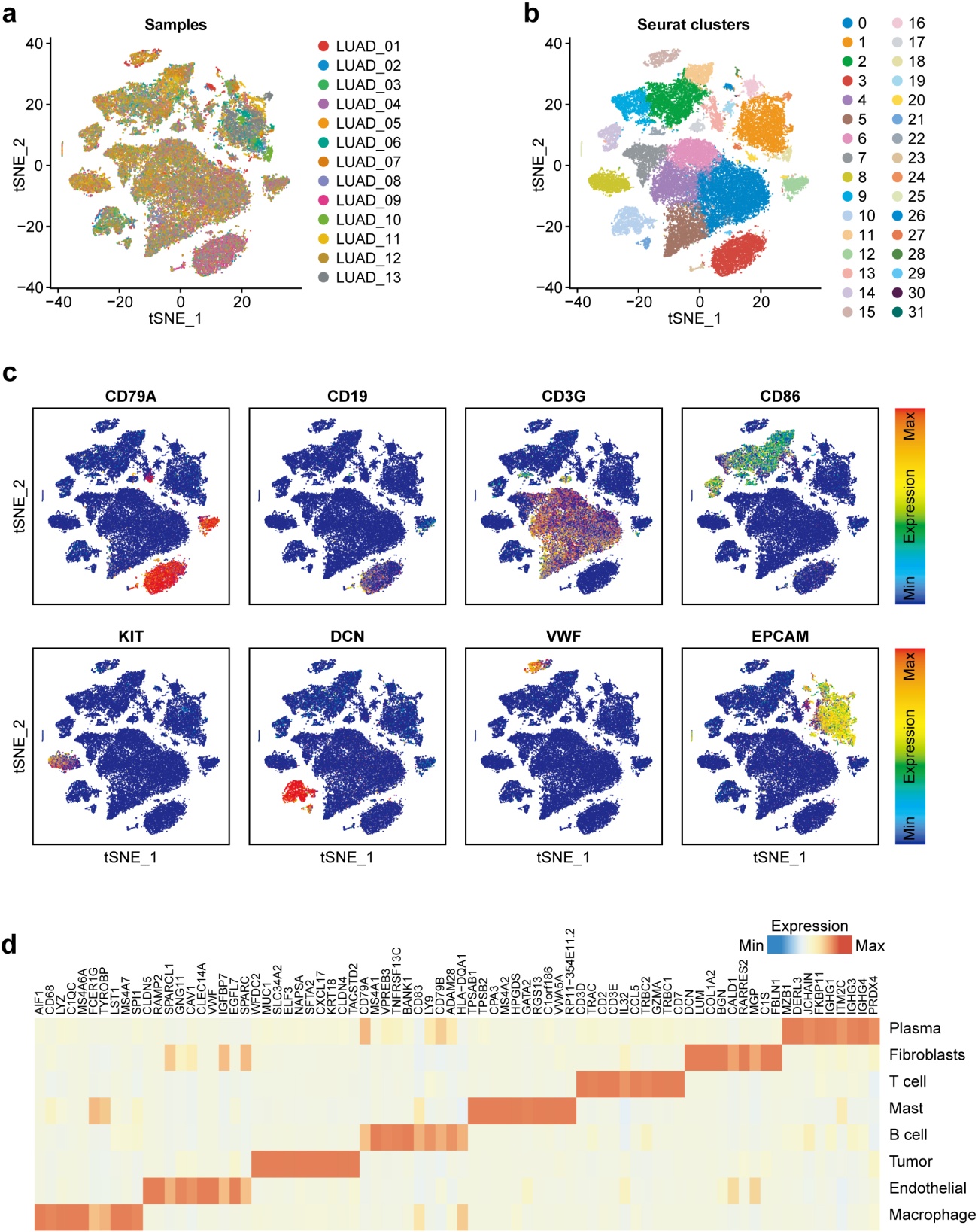
**

**Figure S5. Unsupervised clustering and cell type annotation of scRNA-seq data from 13 LUAD patients.** (a) t-SNE visualization of 46,108 single cells passed quality controls, colored by 13 LUAD samples. (b) The unsupervised clustering of 46,108 cells. (c) Expression levels of cell-type-specific genes overlaid on the t-SNE representation. (d) Heatmap for gene expression levels of top ten cell-type-specific genes.

**
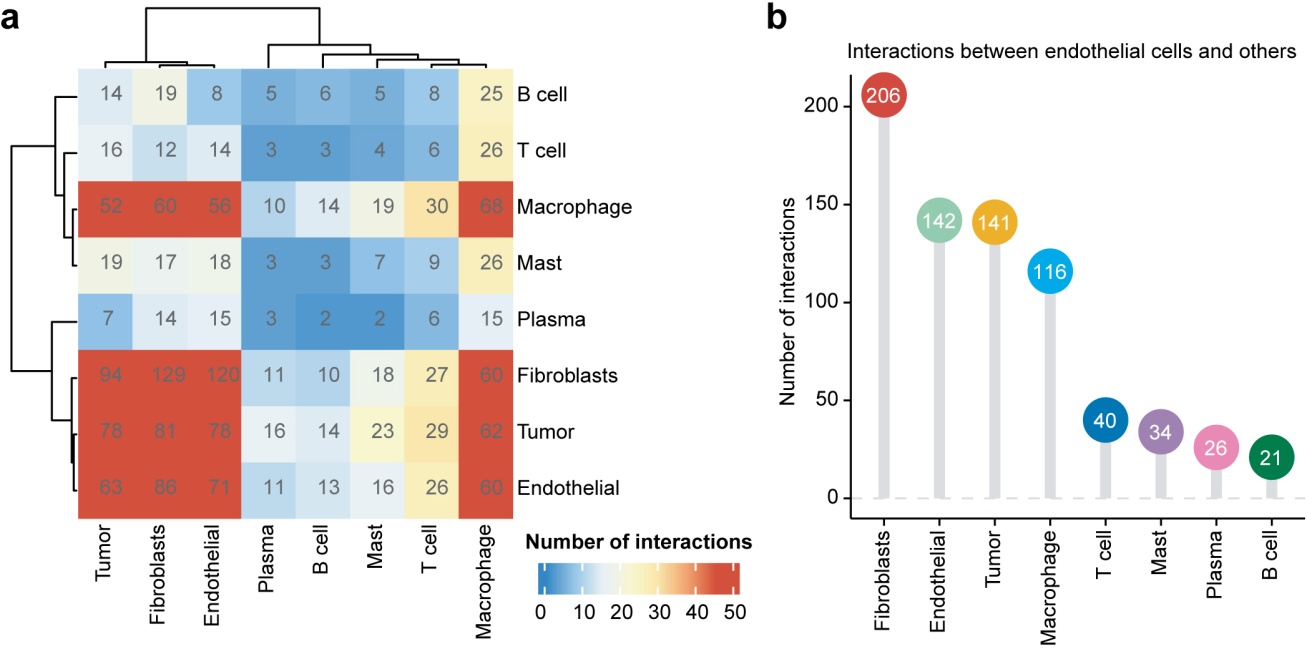
**

**Figure S6. Cell-cell communications among cell types in the scRNA-seq data of 13 LUAD patients.** (a) Heatmap showing the number of interactions among cell types. (b) The number of interactions between endothelial cells and other cells.

**
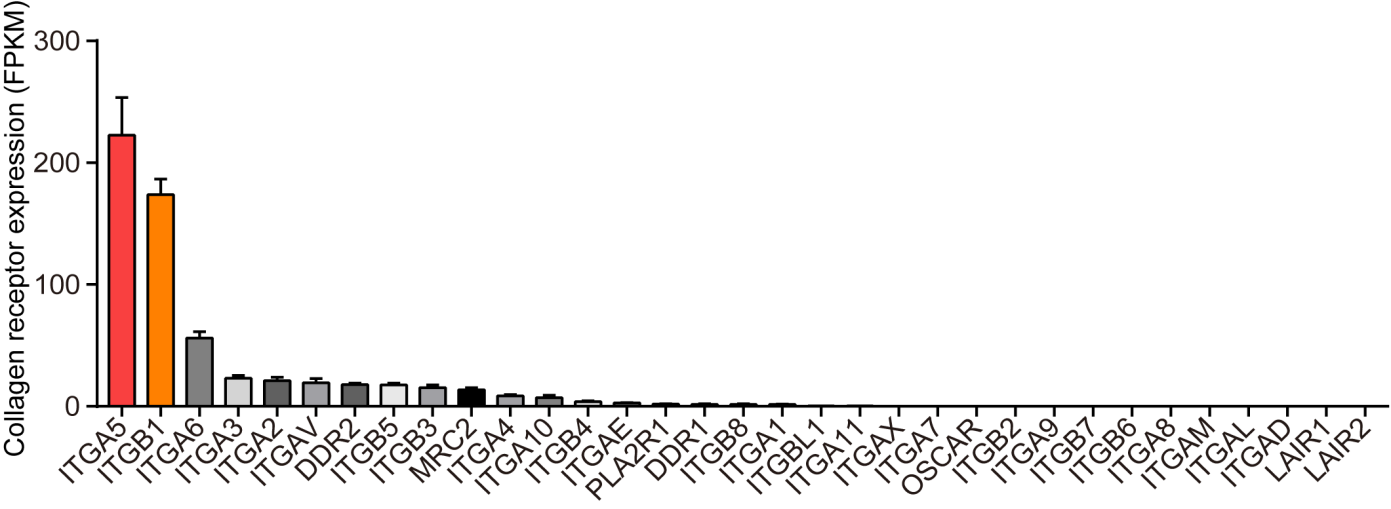
**

**Figure S7. Expression abundance of various collagen receptors in HUVEC cells.** ITGA5 and ITGB1 exhibited the highest abundance. n = 3 per group.

**
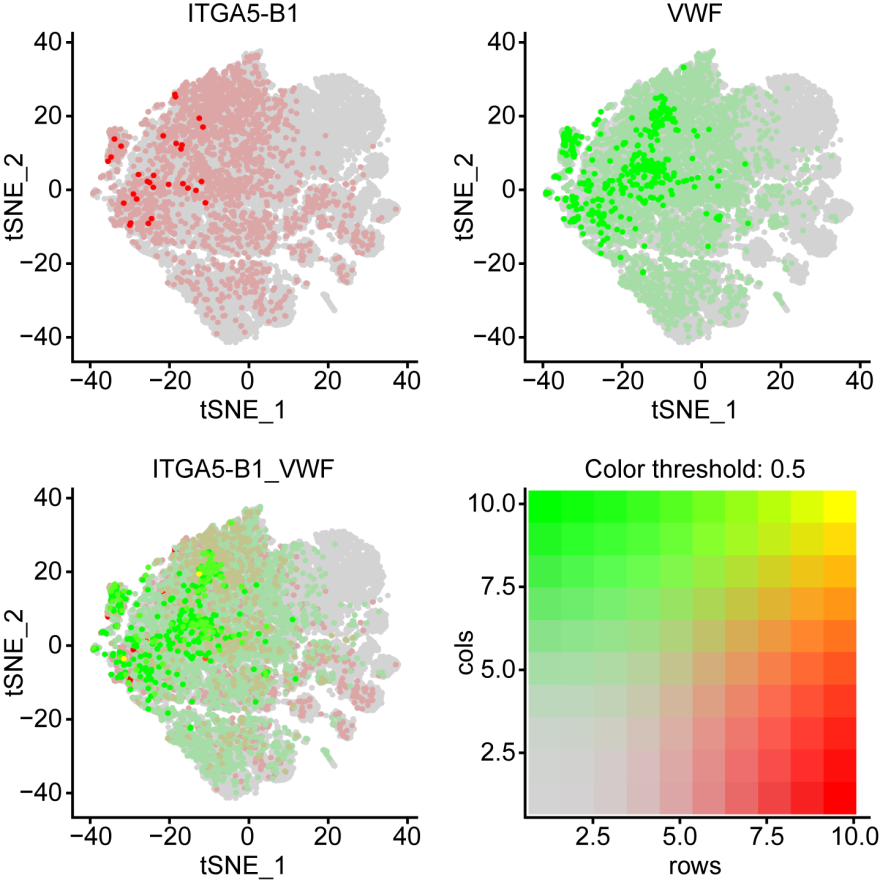
**

**Figure S8. Expression of ITGA5-B1 (gray to red) and VWF (gray to green) on overlaid on the t-SNE representation.** The data was obtained from the spatial transcriptomic dataset.

**
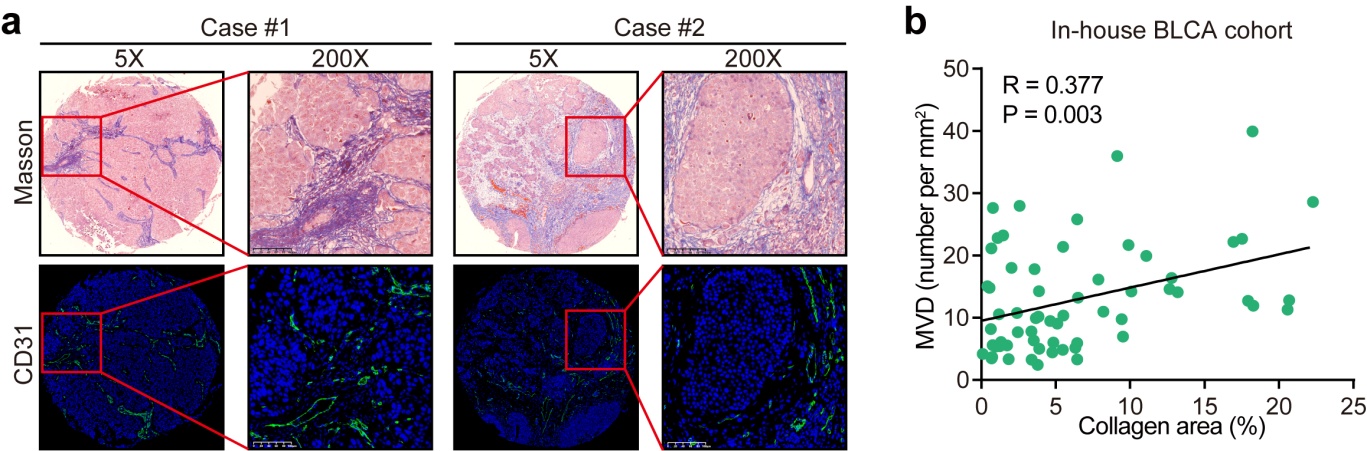
**

**Figure S9. Collagen deposition was positively correlated with the MVD.** (a) Representative images uncovering the distributions of collagen and CD31^+^ endothelial cells in the in-house BLCA cohort. (b) Correlation between collagen levels and the MVD in BLCA samples. Significance was calculated using the Pearson test.

**Table S1. Resources of angiogenesis-related pathways.**

| **Angiogenesis-related items** | **Resources** |
| --- | --- |
| HALLMARK Angiogenesis | https://www.gsea-msigdb.org/gsea/msigdb/human/geneset/HALLMARK_ANGIOGENESIS.html |
| WP Angiogenesis | <https://www.gsea-msigdb.org/gsea/msigdb/human/geneset/WP_ANGIOGENESIS.html> |
| BIOCARTA VEGF pathway | <https://www.gsea-msigdb.org/gsea/msigdb/human/geneset/BIOCARTA_VEGF_PATHWAY.html> |
| WP VEGFA-VEGFR2 signaling | https://www.gsea-msigdb.org/gsea/msigdb/human/geneset/WP_VEGFA_VEGFR2_SIGNALING.html |
| EPIC endothelial cell | https://github.com/GfellerLab/EPIC |
| xCell endothelial cell | https://github.com/dviraran/xCell |

**Table S2. The clinic-pathological parameters of LUAD patients in the in-house therapy cohort.**

| **Parameters** | **C&B arm** | **C&I arm** | **P value** |
| --- | --- | --- | --- |
| Gender |  |  | <0.001 |
| Male | 62 | 69 |  |
| Female | 40 | 11 |  |
| Age | 63.76±9.31 | 65.98±7.67 | 0.088 |
| Response |  |  | 0.926 |
| PR/CR | 26 | 22 |  |
| SD | 63 | 49 |  |
| PD | 13 | 9 |  |
| PFS status |  |  | 0.058 |
| 0 | 25 | 30 |  |
| 1 | 77 | 50 |  |
| Subtype |  |  | 0.539 |
| soft & hot | 29 | 30 |  |
| armored & cold | 45 | 30 |  |
| quiescent | 22 | 14 |  |
| both high | 6 | 6 |  |
